# Context-dependent interactions and the regulation of species richness in freshwater fish

**DOI:** 10.1038/s41467-018-03419-1

**Published:** 2018-03-06

**Authors:** Andrew S. MacDougall, Eric Harvey, Jenny L. McCune, Karin A. Nilsson, Joseph Bennett, Jennifer Firn, Timothy Bartley, James B. Grace, Jocelyn Kelly, Tyler D. Tunney, Bailey McMeans, Shin-Ichiro S. Matsuzaki, Taku Kadoya, Ellen Esch, Kevin Cazelles, Nigel Lester, Kevin S. McCann

**Affiliations:** 1 Department of Integrative Biology, University Of Guelph, Guelph, Ontario Canada N1G 2W1; 2 Institute Of Evolutionary Biology and Environmental Studies, University of Zurich, Ch-8057 Zürich, Switzerland; 3 Department Of Ecology and Evolutionary Biology, University Of Toronto, Toronto, Ontario Canada M5S 3B2; 4 Department Of Biology, Carleton University, Ottawa, Ontario Canada K1S 5B6; 5 Department of Ecology And Environmental Sciences, Umeå University, Umeå, SE-901 87 Sweden; 6 Queensland University Of Technology, Brisbane, Queensland 4000 Australia; 7 US Geological Survey, Wetland And Aquatic Research Center, 700 Cajundome Boulevard, Lafayette, Los Angeles 70506 USA; 8 Fisheries And Oceans Canada, Gulf Fisheries Centre, Moncton, New Brunswick Canada NB EC 9B6; 9 University Of Toronto Mississauga, Mississauga, Ontario Canada L5L 1C6; 10 National Institute For Environmental Studies, Tsukuba, 305-0053 Japan; 11 Ontario Ministry of Natural Resources and Forestry, Peterborough, Ontario Canada K9J 8M5

## Abstract

Species richness is regulated by a complex network of scale-dependent processes. This complexity can obscure the influence of limiting species interactions, making it difficult to determine if abiotic or biotic drivers are more predominant regulators of richness. Using integrative modeling of freshwater fish richness from 721 lakes along an 11^o^ latitudinal gradient, we find negative interactions to be a relatively minor independent predictor of species richness in lakes despite the widespread presence of predators. Instead, interaction effects, when detectable among major functional groups and 231 species pairs, were strong, often positive, but contextually dependent on environment. These results are consistent with the idea that negative interactions internally structure lake communities but do not consistently ‘scale-up’ to regulate richness independently of the environment. The importance of environment for interaction outcomes and its role in the regulation of species richness highlights the potential sensitivity of fish communities to the environmental changes affecting lakes globally.

## Abstract (web summary)

Species richness patterns are driven by biotic and abiotic factors, the relative strengths of which are unclear. Here, the authors test how species interactions or environmental traits influence fish richness across over 700 Canadian lakes, showing a surprisingly small role of negative interactions.

## Introduction

The regulation of species richness reflects complex interactions among local and regional processes, all potentially subject to the influences of global change^1,2^. Of particular importance is the relative strength of limiting species interactions versus abiotic thresholds that determine which species occur in which communities, especially as both factors strongly interact^3–6^. The negative impacts of species interactions on richness derive from a dominant species displacing a subordinate by competition or predation. However, the role that species interactions have in directly and independently determining community membership has been long debated^7–12^. For example, it can be unclear whether communities ‘saturate’ by reaching an upper threshold of richness defined by resource limitations intensifying species interactions^13,14^, the degree to which interaction outcomes are shaped by environmental context that can overwhelm or mask interaction effects in ways difficult to detect^5,6^, and whether interactions are consistently strong enough to regulate the establishment of new species (i.e., biotic resistance)^15–17^.

The uncertainty surrounding species interactions, abiotic factors, and the regulation of local richness persists, because species co-occurrences typically derive from multivariate scale-dependent processes, of which species interactions are likely to be one of many contributing factors^3,4^. Richness regulation in many systems has been variously attributed to climatic gradients, habitat availability, resource levels, regional influences relating to dispersal limitation and spatial isolation, and species interactions within and among trophic levels^3,4,13^. Untangling how these factors combine to shape richness has been limited in part by the traditional use of analytical models that can treat these processes more as competing alternatives than congruent and interacting factors^14,18–20^. Richness regulation may be better understood through analyses that estimate the collective influences of various factors on species occurrences, requiring data representing wide gradients of climate, and multi-level spatial factors such as habitat area and resources^20^.

One of the more widely studied systems for regulation of species richness is freshwater fish, where combinations of regional and local processes including predation and competition affect co-occurrences in complex ways that can be difficult to untangle^21–27^. The importance of limiting species interactions for fish seems axiomatic given the common presence of major predators in most systems but how these antagonistic interactions translate into richness is less certain. Species interactions among fish can sometimes be restrictive on richness, whereas other times positive where predation in particular facilitates coexistence via higher-order interactions^14^. Further, species interactions in lakes can be strongly dependent on environmental context, here defined as when the outcome of predation or competition changes depending on local abiotic conditions. Such context dependency derives in part from inescapable linkages of fish to habitat. Water temperature, e.g., affects fish physiology directly, while shaping how other factors such as nutrients and pH influence trophic dynamics^28–32^. Similarly, lake area, depth, shape, and relative littoral area can variously affect fish richness via influencing water temperature, water quality, and the availability of suitable habitats and resources^14,23,33–37^. The end result is a potentially strong coupling of species interactions and environment, as can occur, e.g., when predatory effects are heightened in shallower and smaller lakes^10,16,17,22,31,32^, or when resource availability influences richness via bottom-up processes^14,37–39^. Given the complex interaction of these various factors, understanding of the regulation of fish richness requires considering multiple factors simultaneously^20,21,40,41^.

Here we use integrative multivariate modeling to examine the relative strength of species interactions alone and combined with climate, lake morphometry, and water quality on among-lake species richness in 721 lakes distributed over an 11° latitudinal gradient in central North America (Ontario, Canada). We test for signals of species interactions by considering two response variables: the numbers of fish species per lake (‘richness’) and co-occurrence of 231 species pairs per lake (hereafter ‘composition’). For the latter, we test for evidence of one species potentially restricting another more often than would be expected by chance, targeting ‘absolute’ restrictions, where two species do not co-occur regardless of lake conditions, and ‘contingent’ restrictions, where two species only co-occur in specific conditions such as deeper and larger lakes^22^. In total, we found negative interactions to be relatively minor independent predictors of species richness even with the widespread presence of major predators. When detected, interaction effects were strong but more likely to be positive than restrictive and almost always intricately coupled with multivariate environmental influences.

## Results

### Overview

**Fig. 1:**
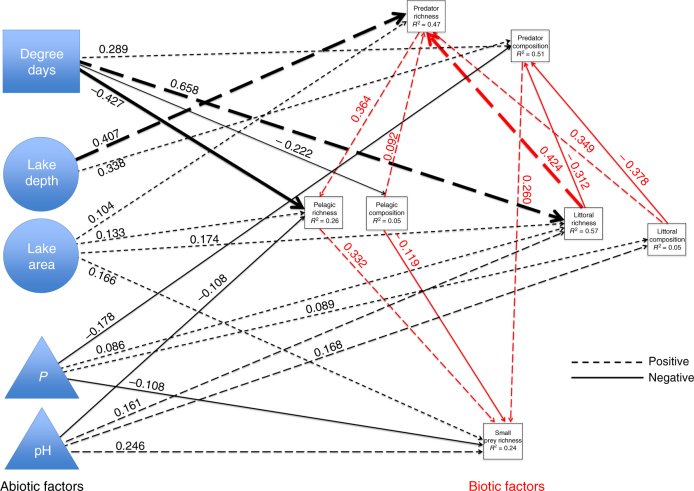
Direct and indirect drivers of species richness in fish. SEM-derived multivariate relationships among integrated abiotic and biotic regulatory factors (blocks = degree days, circles = lake morphometry, triangles = water quality, and biotic factors [red lines]) for the richness and composition of four major fish functional groups in 721 lakes along an 11° latitudinal gradient in Ontario, Canada SEM integrative model:, *n* = 648, ML_EST_ = 4.91, Degree of freedom = 13, *P* = 0.977, see Methods and Supplementary Note 1). Solid lines indicate negative relationships; dashed lines indicate positive relationships. Arrows indicate the direction of the relationship. Bold lines indicate stronger relationships, arbitrarily assigned as standardized path coefficient values > 0.40. Black lines indicate abiotic influences on biotic factors and red lines indicate influences between biotic factors. Functional groups are predator, littoral, pelagic, and small-prey species, with full species list given in Supplementary Table 1

**Fig. 2:**
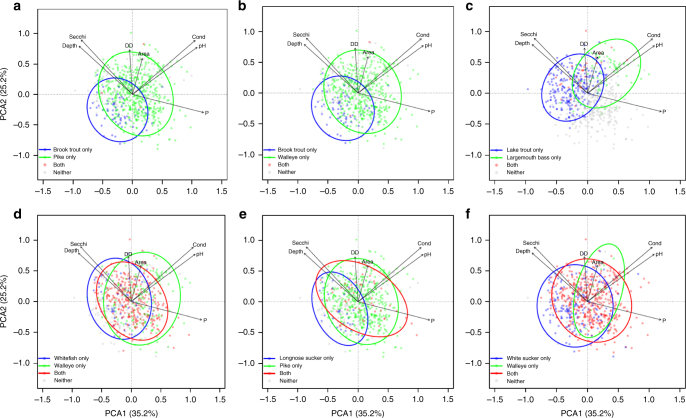
Categories of habitat overlap among fish species. Representative ordinations of lakes in multivariate environmental space for fish species pairs, divided into four broad categories described in Supplementary Table 4: **a**–**c** species rarely co-occur—there can be a large overlap between A alone and B alone (i.e., they can tolerate similar lake conditions), yet they are rarely found together—this is consistent with negative interactions; **d** at least one species inhabits a compressed range of lake conditions when the other is present—this could be due to abiotic limitations of one or both species, such that the range of conditions inhabitable is narrower than when they are alone, or it could result from ‘contingent coexistence,’ where a subordinate can only escape the effects of a dominant in certain environmental conditions; **e** species co-occur quite often with no evidence of a narrowing of environmental conditions in the presence of the other; and **f** one species is found in a greater range of environmental conditions when the other is present. DD = degree days; P = phosphorus; cond = conductivity

**Fig. 3:**
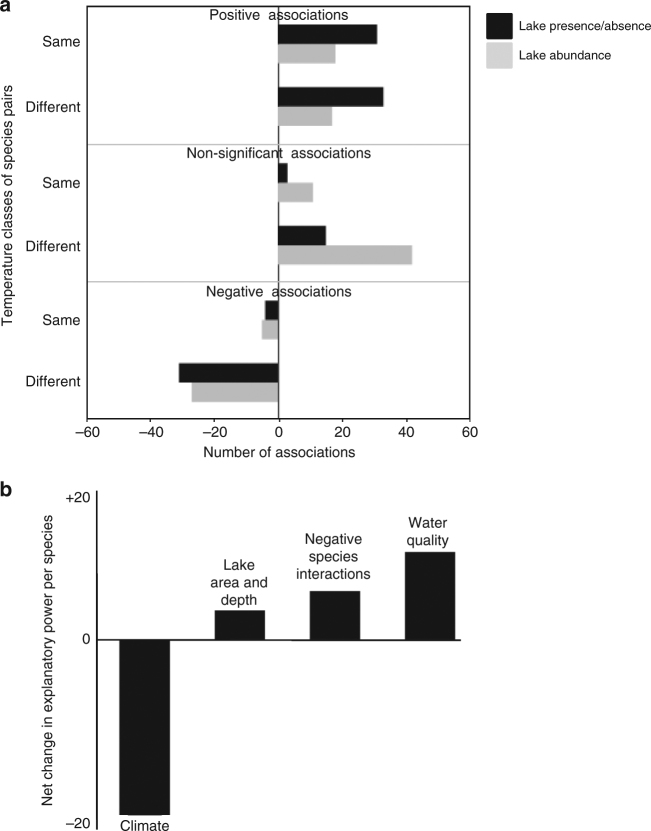
Frequency of negative, positive, and non-associated pair-wise interactions between among-lake richness versus within-lake abundance. **a** Frequency of significantly positive, nonsignificant, and negative associations among 231 species pairs of freshwater fish in 721 lakes, for two classes of data: lake presence/absence (differences in among-lake richness) and lake abundance based on catch per unit effort for each species in each lake (differences in within-lake abundance). We hypothesized an increase in the frequency of negative interactions within-lakes but this was generally not the case, for both species pairs in the ‘same’ temperature class (e.g., two species of ‘warm-water’ fish, Supplementary Table 1) versus ‘different’ classes (warm vs cold-water species). **b** Relative change in the importance of four major explanatory factors (climate, morphometry, water quality, and negative species interactions) between BRT analyses of among-lake richness to BRT analyses of within-lake abundance (see Supplementary Table 3). Significant thresholds were based on an *α* < 0.05

We found limited evidence that species interact strongly enough to consistently restrict one another from lakes, independent of environmental conditions (Figs. 1 and 2, and Supplementary Table 4). Our analyses demonstrate more intricate and complex outcomes where all major factors acted in concert rather than as competing alternatives (Fig. 1 and Supplementary Tables 4, 5). When significant, species interactions were more likely to be positive than limiting (Figs. 1 and 3, and Supplementary Fig. 3).

Each major abiotic and biotic factor that we tested had significant explanatory power (Supplementary Fig. 1). However, none independently explained as much variation, in terms of clarifying direct and indirect linkages among factors, as when integrated together in a single multivariate model (Fig.1 and Supplementary Table 5). We found the influence of species interactions on richness and composition to be largely context-dependent, with the direct effects of one species on another difficult to isolate from the lake conditions where the species occurred (Figs. 1 and 2, and Supplementary Tables 2–4). We observed strong direct abiotic effects on the richness of major functional groups and on species composition in lakes (Fig. 1). We observed significant top-down and bottom-up effects on the numbers of species present in each functional group. Interestingly, these effects tended to increase rather than restrict functional group richness such that more species on one level meant more species on another (Fig. 1 and Supplementary Fig. 4b). Richness thus appears to beget richness in lakes rather than to restrict it. Further, these trophic linkages were again found to be intricately nested in abiotic context. For example, littoral richness was positively associated with predator richness especially in warmer waters (Fig. 1). Finally, we observed significant influences of ‘species composition’ in lakes (structural equation modeling, SEM; Fig. 1 and Supplementary Fig. 3), where the presence of one species was associated with the presence or absence of another. However, as described, few functional group effects could be independently attributed to one species excluding the other, without at least partially considering the influence of abiotic factors such as temperature (Supplementary Tables 2 and 3).

Our finding of limited evidence for exclusion of one species by another was observed despite the widespread presence of aggressive predators such as pike, walleye, and bass (Supplementary Table 1). Of the initial 231 species pairs among the 22 species we examined, only 32 pairs were significantly negatively associated—most species were positively associated or indifferent to the presence of the other (Supplementary Table 2). Of these 32 pairs, only 19 appeared to suggest a role of limiting species interactions based on the species pairs having substantial overlap in environmental affinities yet rarely co-occurring (absolute restriction) or only co-occurring in a reduced subset of lake conditions such as deep, cold, and oligotrophic lakes (contingent restriction; Fig. 2 and Supplementary Table 4). Many of these 19 species pairs involved two salmonids (brook trout, lake trout), with brook trout appearing especially sensitive to predation and competition (Supplementary Tables 2–4).

### Abiotic influences

All abiotic factors were influential on richness and composition, albeit via different pathways (direct and indirect), directions (positive and negative), and magnitudes (Fig. 1). These multivariate influences, unfolding differently by functional group and by species, reinforce how bivariate tests of richness regulation can miss critical factors. The strongest abiotic drivers of species richness were degree days and variables related to lake morphometry (e.g., depth, area; see Fig. 1). The importance of degree days on fish is expected given that they are ectothermic^31,42–45^, although the direct and indirect effects of temperature can be difficult to untangle^36^. Here, direct temperature effects (i.e., growing degree days) had the greatest explanatory power of any factor for among-lake richness while indirectly shaping the influences of other processes (Fig. 1). Lake depth and area had universally positive effects on richness at one or more trophic levels, while indirectly influencing how richness and composition at one trophic level affected other levels. Lake morphometry likely directly affects species interactions (e.g., spatial refugia for prey in larger and deeper lakes^33^), whereas indirectly affecting occurrence patterns as lake area and depth often determine whether lakes thermally stratify (e.g., stratification is critical for lake trout). Lake water quality also affected richness and composition (Fig. 1). pH was significantly associated with the richness of littoral, pelagic, and small prey species, as has been described previously^45^. Unexpectedly, phosphorus levels (P) did not vary by latitude despite human populations being concentrated to the south (Supplementary Figs. 1 and 2). There were also interactions among abiotic factors, such as larger-sized and higher-pH lakes having more total P (Supplementary Fig. 2). Again, these complex interactions illustrate that integrative multivariate approaches are crucial for determining the regulation of species richness.

### Trophic interactions

Bottom-up and top-down biotic linkages also varied in magnitude, especially when comparing connections among the richness of major functional groups versus the species composition of those groups (Fig. 1). For functional group richness, the highest interaction-based explanatory power was for predators (*R*^2^ = 0.47), which was positively associated with higher littoral richness and higher richness and composition of pelagic species (Fig. 1). Such ‘bottom-up’ positive effects from prey to predators, with relatively high explanatory power, are suggestive of trophic dependencies with the richness of the latter group dependent on the former for persistence^46,47^. Stated again, antagonistic species interactions are clearly detectable in our system yet appear to act more to promote than suppress richness. Further, there were strong contextual influences on these biotic dependencies, with littoral richness and pelagic composition –both important for predators as described - regulated significantly by abiotic factors. All significant top-down influences of predator richness on richness lower in the food web were also positive. Our analysis cannot determine whether these positive ‘top-down‘ associations mechanistically reflect shared abiotic drivers promoting higher richness in all functional groups, some degree of trophic facilitation where predation promotes prey co-coexistence, or both. Regardless, our results support the predicted importance of bottom-up and top-down biotic interactions for trophic structure in lakes, with the impacts of both shaped more by environmental context than operating independently.

### Pair-wise interactions

Although associations among the richness of functional groups were mostly positive, the composition of those groups was a mixture of positive and negative associations (Figs. 1 and 2, and Supplementary Fig. 3). Using principal component analyses (PCA) associated with the SEM (see Methods), we identified 16 of the 22 species to be significantly associated with the presence of another—we interpreted these to potentially suggest interactions among species pairs that could affect co-occurrence. That being noted, the causal connections cannot be identified by the SE model—associations among species whether significantly positive or negative can derive from shared versus divergent environmental affinity or positive versus negative species interactions^48^.

Our three additional analyses were used to address the problem of separating habitat affinity versus interactions on species composition, especially for negatively associated pairs. Our results demonstrate that these two possibilities—negative associations by affinity versus negative associations by antagonistic interactions—represent two extremes of a gradient along which each of the 32 negatively associated species pairs appear to be uniquely positioned (Fig. 2 and Supplementary Table 4). Some pairs rarely co-occurred despite substantial habitat overlap (e.g., brook trout with pike and walleye). We interpreted these pairs as the most suggestive of absolute restrictions. Some pairs such as walleye and whitefish often co-occurred but in a significantly narrower set of environmental conditions than where they occurred alone (i.e., contingent restrictions). Other negative associations appeared to be mostly attributable to divergent habitat affinity based on limited habitat overlap (e.g., longnose and white sucker, with pike and walleye). Almost all of these 32 negative associations—absolute, contingent, or attributable to divergent habitats—involved species pairs of different temperature classes (cold versus warm water fish), again suggesting the influence of environment on interaction outcomes (Supplementary Tables 2 and 3).

### Richness vs abundance analysis

Our additional analyses using species abundance data did not fundamentally change our results—we observed small increases in the frequency of negative relationships among species pairs but abiotic factors remained predominant. For the correlation analyses among species pairs (Fig. 3a) the biggest change was a large decrease in the number of positive species pairings toward more nonsignificant associations. The reasons for this change are suggested by the boosted regression tree (BRT) analyses testing the primary drivers of lake abundance for each species, with a 50% decrease in the explanatory power of climate (Fig. 3b).

## Discussion

Our analysis is one of the more comprehensive empirical tests of the importance of limiting species interactions for regulation of richness and composition, using integrative analyses and fish data from 721 lakes covering large gradients of degree days, latitude, lake morphometry, water quality, and trophic complexity. Although we detected evidence of significant influences of species interactions on diversity and composition, negative interactions in isolation were unable to consistently predict co-occurrences along multiple regional-scale environmental gradients—fish appear to rarely forbid one another from lakes. This conclusion does not indicate that antagonistic species interactions are unimportant for the regulation of fish communities, rather it implies that it is difficult to detect independent signals of their influence on species richness among lakes. Indeed, dozens of studies have explored richness regulation in fish, driven by interest in both fundamental ecological mechanisms and fisheries management^21–37,41^. Our work provides clarification on why these questions have been difficult to test, with richness regulated by complex multivariate factors that operate at a range of spatial resolutions.

Our findings were robust, with similar results for the presence-absence of species pairs among lakes versus the analysis of species abundance within lakes. The only factor to significantly change was the role of climate, which had stronger impacts on among lake differences as would be expected given the strong sensitivity of fish to water temperature (e.g., cold-water species tend to be positively associated with each other, in cold water lakes). Once species pass this climate filter, however, within-lake processes relating to lake depth, lake size, water quality, and, to a smaller degree, negative interactions appear to become more influential. Further, these non-climatic factors appear to vary more uniquely for each species, such that the number of positive associations within lakes becomes reduced (e.g., within any cold-water lake, one cold-water species may be littoral while another pelagic; thus, their associations are no longer positive at this finer scale).

The failure to detect significant increases in negative associations with the abundance data points to the influence of spatial scale on interaction outcomes that even analyses of abundance per lake were not fine enough to detect strong signals of antagonistic interactions. The most likely explanation is that the influence of limiting species interactions occurs at finer scales than we tested. Considerable work has shown the importance of antagonistic interactions at finer scales in lakes, e.g., between near-shore and open water habitats^36,41^. Whether such localized interactions ‘scale up’ to affect and abundance in an entire lake can again depend on environmental context. For example, the predatory effects of lake trout on prey are mediated by the availability of cold refuge spots, as lake size decreases and temperature increases^36^. This is consistent with our results: whether interactions strongly affect total lake richness depends on how lake conditions weaken or intensify how one species affects another. These considerations suggest that the regulation of species is best detected by multifactor analyses, supporting a hierarchical scale-dependent channeling of richness with biotic factors more influential at finer scales.

Our finding of powerful multi-factor abiotic and environmentally dependent interaction effects begs the question of generality to other systems, including other freshwater lakes where top-down forces are known to be limiting^48,49^. Foremost, other studies documenting top-down effects have also detected context-dependency relating to lake depth, lake area, temperature, and scale^10,25,31,42,50^. In addition, our data capture a wide range of interacting gradients in hundreds of lakes, yet other systems may simply have different profiles of environmental complexity. Our gradient of lake area, for example, extends over three orders of magnitude, yet the mean area of these post-glacial lakes is relatively large by global standards (325 ha). Our larger lakes may thus contribute to the higher explanatory power of abiotic factors, whereas direct and independent predatory influences may be more pronounced in systems where lakes are smaller and shallower^22,33,49^. In total, these considerations again emphasize how environmental context may affect the strength of limiting species interactions, including the possibility that their influence differs among lake systems globally.

Perspectives on the power of species interactions for regulating species richness have varied, from maintaining coexistence to being relatively weak compared with abiotic drivers^3,4^. Our integrative work shows why such uncertainties can persist, with the direct independent influence of species interactions on richness difficult to detect in a system where predation and competition are known to be strong^36^. These results highlight how the strength of species interactions can be strongly coupled to abiotic conditions^32,36^, indeed masking their impacts at least at the scales tested here. Finally, the importance of abiotic factors for the richness of freshwater fish reinforces the potential sensitivity of lakes to environmental change. Freshwater covers < 1% of the earth’s surface, while supplying 12% of the fish consumed by humans^51,52^. Our work shows how these economically valuable fish communities can be significantly affected by abiotic drivers and by implication be sensitive to environmental changes affecting lake conditions including climate warming and eutrophication^50,52^. These considerations illustrate how the integrative approaches used in our study can help clarify fundamental mechanisms on richness regulation, while revealing how these mechanisms can be anthropogenically transformed.

## Methods

### Data

Our sampled lakes occur across an area of 450,000 km^2^ and capture gradients of climate, lake morphometry, water quality (e.g., total P, pH), and trophic richness with varying numbers of predatory, littoral, pelagic, and small-prey fish species (Supplementary Table 1). The regulation of richness for fish has been tested previously in some Ontario lakes, with each study demonstrating the importance of one or more these regulatory factors^14,16,22–30,36,37^. None, however, have fully examined their relative direct and indirect (i.e., interacting significantly with other factors) influences on fish distributions, although the need for such an approach has been acknowledged^22^. Further, regional impacts on local richness can be significant, potentially overwhelming finer scale abiotic and biotic processes on community assembly in ways that can be difficult to test^4,25^. In our system, however, these processes relating to latitudinal isolation and dispersal limitation are thought to be influential but secondary relative to local factors^40^ and weaker than typical for lake systems globally^25–27^. The main reason reflects long-term dispersal dynamics. Following glacial retreat in the late Pleistocene, many Ontario rivers initially flowed north before eventually shifting south, such that all of our targeted species dispersed and occur across all or most of the latitudinal gradient at least to the extent of their temperature thresholds (e.g., warm-water fish such as large-mouth bass are absent in the most northerly lakes). A second reason reflects the likely homogenizing influence of introductions of native species by humans over the last century. Although none of our fish populations are maintained by introductions, numerous native species have been added to lakes intentionally or accidently (e.g., small-prey bait fish). Although the exact magnitude of these additions are unclear, especially their degree of success (many introductions may fail), they would serve to further reduce regional-based influences of dispersal limitation on lake-level species richness^10,16^. In total, the relative weakness of regional influences means our data provide a unique opportunity to focus squarely on localized lake-level abiotic and biotic drivers of species richness.

The lake data derived from the Province of Ontario’s Broad-scale Monitoring (BsM) fish database, with all lakes surveyed for species richness (number of fish species per lake), species identity, numbers of fish per species per lake based on standardized netting protocols among all lakes, and a range of abiotic and lake morphological data. Details of sampling intensity, timing, and fish netting procedures can be found in Sandstrom et al.^53^. In short, the BsM sampling protocols generally resemble standardized methods in Europe and elsewhere in North America, with the BsM using two mesh sizes for capturing fish of different size classes^53^. We extracted four classes of data from the BsM data, relating to our regulatory models of climate (degree days), lake morphometry (area, mean, and maximum depth), lake water quality (P, pH, and for some analyses conductivity and Secchi depth), and species interactions. Classification of fish functional groups followed Holm and Mandrak^54^. Fish richness ranges from 1 to 24 species per lake, out of a total pool of 65 species in the BsM data—our work only dealt with 22 of these 65 species (see Methods and Supplementary Notes). These 22 species had larger body sizes (mean length > 10 cm) and were found in a minimum of 40 of the 721 lakes (Supplementary Table 1). There were an additional 15 larger body species found in < 40 lakes. These infrequent species could be more sensitive to species interactions and thus more likely to be displaced by limiting species interactions, but this could not be tested due to reduced statistical power relating to their rarity. We also pooled 28 additional species of the 65 into one functional class (“small prey species;” Supplementary Table 1); these species have smaller body sizes (mean length < 10 cm), tend to be mostly littoral species, and are likely to be consumed by predatory species. This grouping also served a methodological purpose, as small prey species were detected in hundreds of lakes but may be under-sampled by the BsM netting surveys even with their use of smaller mesh-sized nets^53^.

### Analysis

Our analysis sequentially integrates four complementary procedures. We first used SEM to quantify the interaction pathways among fish functional groups, climate, lake morphometry, and water quality (see Methods and Supplementary Note 1 for procedures and interpretations). This analysis included quantifying the importance of shifts in species composition, where compositional shifts at one trophic level explain compositional shifts at another. To further disentangle the relative contribution of abiotic versus biotic associations as main drivers of fish richness and composition, we also compared the explanatory power of the fully integrative SEM to alternative scenarios with the same variable structure but different directions of effect among functional groups (i.e., top-down effects of predators on prey, bottom-up effects of prey on predators, and abiotic effects with no associations among functional groups; Supplementary Fig. 4).

Next, we examined patterns of co-occurrence among the 231 species pairs using three additional analyses. These analyses tested whether co-occurrence relates more to habitat affinity (e.g., species *x* present because of water temperature), antagonistic species interactions (species *x* present because species y absent), or the context-dependent interaction of the two (e.g., species *x* present because species *y* absent but only in colder waters). We started with null-adjusted Pearson’s correlation analyses to determine the direction of association within lakes—positive, negative, or insignificant—among species pairs (Supplementary Table 2). For the subset of negatively correlated pairs, we then used ANOSIM and PERMDISP tests to examine levels of habitat overlap between each pair. The overlap analyses worked by contrasting environmental factors among four different combinations of lakes: lakes with species *x* and *y* co-occurring, lakes with either species *x* or species *y* alone, and lakes with neither. We could then test whether, for example, species *x* occurs in a wider range of lake conditions when species y is absent. Finally, we used BRT to quantify the relative explanatory power of abiotic and biotic factors for each species separately (Supplementary Table 3).

Our primary focus was to detect evidence of species displacement (species *x* absent when species *y* present) from lakes by predation or competition, after accounting for environmental influences and chance. However, there is the possibility of scale-dependent effects of species interactions, with impacts more directly detectable at finer spatial scales than the lake-by-lake comparisons of our main analysis. That is to say, species *x* and *y* may easily coexist in lakes but the latter may be significantly rarer when the other is present. To test for this, we conducted finer-scale analyses of fish population sizes within all of our 721 lakes. We used supplementary correlation and BRT analyses on catch-per-unit-effort ‘abundance’ data describing the numbers of individuals per catch effort for each species in each lake. We then contrasted these results with the same analysis on the among-lake occurrence data, to see whether the frequency and explanatory power of negative species associations intensified for finer-scale abundance patterns within lakes.

We used a four-tiered analysis centered on SEMs to test multivariate interactions among the four overarching models of richness regulation, constructing a multivariate meta-model^20^ that explored hypothesized interactions among climate, lake morphometry, water quality, and consumptive- and competitive-based species interactions. We simplified the SEM by eliminating several factors that were highly correlated—e.g., total phosphorus was used to represent productivity-based water quality, given its close correlation with measures of conductivity, and Secchi depth. Species interactions in the SEM were represented by two components. The first was the total number of species per lake in four functional groups—predators, pelagic, littoral, and small prey fish (i.e., does the richness of species in one group relate to the richness of another?). The second component of species interactions was the sensitivity of changes in species composition per lake, specifically whether changes in the identity of species in a functional group significantly affected species richness or species occurrence in the other groups. For example, predator richness was significantly and positively affected by the ‘compositional sensitivity’ measure of littoral species (shifts from lakes with the littoral species ‘rock bass’ and ‘yellow perch’ [+ association on predator richness] to those with white suckers and brown bullhead [– association]), meaning that predator richness varied depending on which littoral species were present. The direction of these responses, positive or negative, could reflect potential ‘interaction hotspots’ within the SEM (e.g., predator richness being significantly affected by availability of littoral prey). However, these responses could also reflect negative habitat affinities if some predator species are less suited to the lake conditions that some prey prefer.

To assess compositional shifts in the SEM we used principal component analysis on Hellinger transformed data^55^ for each functional level. For predators, only PCA1 was found to be significant during the iterative process of building the SEM model. Predator PCA1 explained 40% of the variance and represented a gradient from pike and walleye to burbot and lake trout dominated lakes (Supplementary Fig. 3). For littoral and pelagic fishes, PCA1 was found in both cases to be strongly correlated with fish richness at each functional group (80% and 75%, respectively). Because littoral and pelagic richness are already included into the SEM, it would have been redundant to have both PCA1 and richness for those two functional groups. Moreover, we wanted our PCA axes to represent compositional shifts independent of changes in richness. For those reasons we kept PCA2 only to represent littoral and pelagic fish composition. PCA2 for littoral fish explained 50% of the variance and represented a gradient from lakes dominated by white sucker, longnose sucker, brown bullhead, and pumpkinseed to those dominated by yellow perch, rock bass and shorthead redhorse (Supplementary Fig. 3). PCA2 for pelagic fish explained 80% for the variance represented a gradient from cisco- to whitefish-dominated lakes (Supplementary Fig. 3).

Our SEM analysis focused on the hypothesis that an integrated model capturing abiotic and biotic interactions within and among functional groups would best capture the regulation of species richness^22^ (see Supplementary Note 1 for a detailed description of our approach). Using an iterative model building approach, we first constructed an ‘integrative’ model capturing the combined effects of abiotic and biotic (top-down and bottom-up associations among functional groups) factors in shaping species richness (Fig. 1). We then constructed more focused and streamlined singular models, which reflect evidence in the fish literature of richness regulation by (I) top-down regulation by major predators, (II) bottom-up models where species richness in lower levels of the food chain influences richness levels above, and (III) abiotic-based models where climate and resources affect productivity, and thus richness, at all levels. We compared the relative explanatory power of these models with each other, and against the fully integrative model using Akaike Information Criteria (see Supplementary Table 5 for model comparison).

Of the 22 species examined, our SEM-related PCA analyses detected 16 that were associated with potential ‘interaction hotspots’ based on the influence of composition (the presence of one or more of these species) on the richness of other functional groups or the occurrence of other species (Supplementary Fig. 3). We used three subsequent analyses to test the direction of association of all possible pair-wise interactions among these 16 species (positive, negative, or none detectable). For those associations identified as negative, we then tested whether there was evidence for interaction-mediated ‘absolute’ or ‘contingent’ restrictions, non-interactive divergent habitat affinity, or a combination of the two.

We used association tests to judge the direction of relationships (negative, positive, non-significant) among each species pair, adjusted with null models derived from Pearson’s correlation coefficients to account for the positive or negative associations occurring as statistical artefacts (Supplementary Table 2^56,57^). The null correction uses a randomization procedure to determine and correct for the null expected correlation in the data, which can be > 0, because double zeros can contribute to positive correlations^56^. Of the all-possible pairings, 53% showed no significant association, whereas 28% were significantly positively associated (i.e., species *x* positively associated with species *y*, greater than expected by chance). We removed these species pairings from subsequent analyses, thereby concentrating on the remaining 32 species pairs that were significantly negative (Supplementary Table 2) and most likely to reflect antagonistic species interactions.

We next used multivariate tests of environmental niche overlap and dispersion (ANOSIM and PERMDISP^58–60^), to examine the distribution in multivariate environmental space of three of the four lake profiles for each the 32 species pairs: lakes where both species are present (co-occurrence) and lakes where only one species or the other is present (Supplementary Table 4). We do not report the results of the fourth combination type—species both absent—although this almost always involved lakes too small, warm, and shallow to consistently support any of the 22 species that we examined. ANOSIM tested for differences (habitat overlap) in the multivariate conditions of lakes types where the two species in each pair occur in isolation from one another. PERMDISP tested for differences in the multivariate environmental variation or range (‘compression’) among the lake types, specifically whether co-occurrence was restricted to a smaller range of lake conditions compared with where the species occurred with the other absent. Together, these analyses allowed us to test for evidence of two potential types of species restrictions —‘absolute’ where two species have considerable environmental niche overlap in the lakes where they occur without the other, yet rarely co-occur, and ‘contingent’ where two species commonly co-occur but only under reduced sets of environmental conditions. A contingent restriction can describe conditions where one species excludes another, but only under specific environmental conditions such as when lakes become smaller, shallower, warmer, or more eutrophic^11^.

Finally, we used BRTs to explore the explanatory power of all major abiotic and species-level influences on each of the negatively associated species pairs^61^. This was necessary because the ‘absolute’ and ‘contingent’ combinations can also be explained or influenced by abiotic factors—e.g., co-occurrence could be restricted to subsets of lakes (our definition of ‘contingent’ restrictions), because few lakes happen to have environmental conditions suitable for both species, and which may or may not interact with competition or predation. The BRTs allowed us to assess the relative explanatory power of abiotic factors and the presence of all of the other species on the occurrence of each species individually, in such cases (Supplementary Table 3 and Supplementary Fig. 5).

### Data availability

The data were collected through Ontario’s BsM program and are available upon reasonable request to the Aquatic Research and Monitoring Section, Ontario Ministry of Natural Resources and Forestry, 2140 East Bank Drive, Peterborough, Ontario, Canada K9J 7B8.

## Electronic supplementary material

Supplementary Information
