# Supplementary material for: Context-dependent interactions and the regulation of species richness in freshwater fish
Source: Nat Commun. 2018 Mar 6;9:973. doi: 10.1038/s41467-018-03419-1 (PMC5840330; doi:10.1038/s41467-018-03419-1)
Supplement: Supplementary file 1 — Supplementary Information [file 41467_2018_3419_MOESM1_ESM.pdf]

Context-dependent interactions and the regulation of species richness in freshwater fish

MacDougall, A.S. et al.

Supplementary Table 1. Species list of (A) Ontario fish species included in this study, based on species captured by BSM surveys in at least 40 of the 721 lakes, and (B) species grouped into the category of 'small prey': these species are assumed to be prey for most other fish species, and mostly inhabit the littoral zone of lakes. Further, their small size means they tend to be under-sampled by the BSM net-based fish sampling. The remaining species (C) occurred in fewer than 40 lakes, and were excluded from the analysis due to their infrequency. The 721 lakes occur over an 11-degree latitudinal gradient (43.06 – 54.52 degrees), range in area from 21-90,484 ha, have maximum depths of 1-214 m, range in phosphorus levels from 1.7-52.5 units, and have a range of total degree days from 1059-2246. Average length for each species are from Ontario lakes, and taken from Holm et al (2010)[1]. Thermal guilds (water temperature preferences) are taken from Hasnain et al. 2013[2].

Common Name	Species name	Family	Number of lakes (n = 721)	Average length (cm)	Thermal guild	Functional Group
(A) *Large fishes species*						
Northern Pike	*Esox lucius*	Esocidae	528	61.0	Cool	Predator
Muskellunge	*Esox*	Esocidae	43	96.5	Warm	Predator

	masquinongy					
Walleye	*Sander vitreus*	Percidae	499	42.0	Cool	Predator
Lake Trout	*Salvelinus namaycush*	Salmonidae	298	44.5	Cold	Predator
Burbot	*Lota lota*	Gadidae	250	38.0	Cold/Cool	Predator
Cisco	*Coregonus artedi*	Salmonidae	385	25.0	Cold	Pelagic
Lake Whitefish	*Coregonus clupeaformis*	Salmonidae	374	38.0	Cold	Pelagic
Brook Trout	*Salvelinus fontinalis*	Salmonidae	77	28.0	Cold	Pelagic
Longnose Sucker	*Catostomus catostomus*	Catostomidae	82	33.5	Cold	Littoral
White Sucker	*Catostomus commersonii*	Catostomidae	678	41	Cool	Littoral
Shorthead Redhorse	*Moxostoma macrolepidotum*	Catostomidae	90	41.0	Warm	Littoral
Rock Bass	*Ambloplites rupestris*	Centrarchidae	295	20.0	Cool	Littoral
Pumpkinseed Sunfish	*Lepomis gibbosus*	Centrarchidae	154	18.0	Warm	Littoral
Bluegill	*Lepomis macrochirus*	Centrarchidae	48	19.0	Warm	Littoral
Smallmouth Bass	*Micropterus dolomieu*	Centrarchidae	333	30.0	Warm	Predator
Largemouth	*Micropterus*	Centrarchidae	85	30.0	Warm	Predator

Bass	*salmoides*					
Black Crappie	*Pomoxis nigromaculatus*	Centrarchidae	55	21.5	Cool	Littoral
Brown Bullhead	*Ameiurus nebulosus*	Ictaluridae	133	28.0	Warm	Littoral
Yellow Perch	*Perca flavescens*	Percidae	526	18.0	Cool	Littoral
Sauger	*Sander canadensis*	Percidae	45	33.0	Cool	Predator
(B) *Small prey fishes*						
Mottled Sculpin	*Cottus bairdii*	Cottidae	51	7.5	Cold	Small prey
Slimy Sculpin	*Cottus cognatus*	Cottidae	51	7.5	Cold	Small prey
Spoonhead Sculpin	*Cottus ricei*	Cottidae	22	5.5	Cold	Small prey
Deepwater sculpin	*Myoxocephalus thompsonii*	Cottidae	11	8.0	Cold	Small prey
Lake Chub	*Couesius plumbeus*	Cyprinidae	104	10.0	Cold	Small prey
Common Shiner	*Luxilus cornutus*	Cyprinidae	155	9.0	Cool	Small prey
Northern Pearl Dace	*Margariscus margarita*	Cyprinidae	50	9.0	Cold/cool	Small prey
Golden Shiner	*Notemigonus crysoleucas*	Cyprinidae	8	10.0	Cool	Small prey

Emerald Shiner	*Notropis atherinoides*	Cyprinidae	159	7.5	Cool	Small prey
Blackchin Shiner	*Notropis heterodon*	Cyprinidae	49	6.0	Cool/warm	Small prey
Blacknose Shiner	*Notropis heterolepis*	Cyprinidae	127	6.5	Cool/warm	Small prey
Spottail Shiner	*Notropis hudsonius*	Cyprinidae	344	7.0	Cold/cool	Small prey
Mimic Shiner	*Notropis volucellus*	Cyprinidae	46	6.0	Warm	Small prey
Northern Redbelly Dace	*Phoxinus eos**	Cyprinidae	42	5.5	Cool/warm	Small prey
Bluntnose Minnow	*Pimephales notatus*	Cyprinidae	113	6.5	Warm	Small prey
Fathead Minnow	*Pimephales promelas*	Cyprinidae	42	5.0	Warm	Small prey
Blacknose Dace	*Rhinichthys atratulus*	Cyprinidae	13	8.0	Cool	Small prey
Longnose Dace	*Rhinichthys cataractae*	Cyprinidae	12	7.5	Cool	Small prey
Creek Chub	*Semotilus atromaculatus*	Cyprinidae	39	10.0	Cool	Small prey
Banded Killifish	*Fundulus diaphanus*	Fundulidae	9	7.0	Cool	Small prey
Brook	*Culaea*	Gasterosteidae	40	5.0	Cool	Small prey

Stickleback	*inconstans*					
Ninespine Stickleback	*Pungitius pungitius*	Gasterosteidae	64	6.5	Warm/cold*	Small prey
Iowa Darter	*Etheostoma exile*	Percidae	13	5.0	Cool	Small prey
Johnny Darter	*Etheostoma nigrum*	Percidae	39	5.0	Cool	Small prey
Logperch	*Percina caprodes*	Percidae	180	9.0	Cool/warm	Small prey
Trout-perch	*Percopsis omiscomaycus*	Percopsidae	302	9.0	Cold	Small prey
Central Mudminnow	*Umbra limi*	Umbridae	9	7.5	Cool/warm	Small prey
(C) *Less common species*						
Longnose gar	*Lepisosteus osseus*	Lepisosteidae	10	76.0	Warm	Predator
Alewife	*Alosa pseudoharengus*	Clupeidae	5	15.0	Cold	Pelagic
Goldeye	*Hiodon alosoides*	Hiodontidae	6	28.0	Warm	Pelagic
Mooneye	*Hiodon tergisus*	Hiodontidae	13	28.0	Cool/warm	Pelagic
Rainbow trout	*Oncorhynchus mykiss*	Salmonidae	4	53.0	Cold	Pelagic
Round whitefish	*Prosopium cylindraceum*	Salmonidae	12	25.0	Cold	Pelagic
Bowfin	*Amia calva*	Amiidae	9	54.0	Warm	Littoral

Silver Redhorse	*Moxostoma anisurum*	Catostomidae	21	40.0	Cool	Littoral
Greater Redhorse	*Moxostoma valenciennesi*	Catostomidae	10	41.0	Cool/warm	Littoral
Common Carp	*Cyprinus carpio*	Cyprinidae	14	37.0	Warm	Littoral
Fallfish	*Semotilus corporalis*	Cyprinidae	13	20.0	Cool	Littoral
Yellow Bullhead	*Ameiurus natalis*	Ictaluridae	20	25.5	Warm	Littoral

Supplementary Table 2. Species association values, measured as null-adjusted Pearson correlation coefficients for abundances of 16 species identified as potential ‘interaction’ species by the SEM analysis (Figs. 1, Suppl. Fig. 3). The association analysis tests the direction and statistical significance of these ‘interactions’, correcting for bias in zero-inflated data arising because the correlation coefficient is not double-zero symmetrical[3]. Significantly negative (32/120) pairings are grey or black highlighted cells. These results cannot test whether the significant associations derive from species interactions, or if negative associations derive from divergent habitat affinity (e.g., species x is absent when species y is present, because they prefer different lake conditions). However, by organizing these results by temperature affinity (BLUE = cold water species, RED = cool, or GREEN = warm), most negative interactions occur among temperature groups (grey cells) – for example, warm and cold water species tending not to co-occur. Only five negative species pairings were within- group (black cells). There were 37/120 positive pairings and 51/120 non-significant pairings – we assumed these associations to not reflect antagonistic species interactions, and thus could not generate restricted co-occurrence between the species pairs.

Species	LNSk	Whtfh	BTrt	Burb	Cisco	YPer	Pike	Wall	WSk	Red	SMB	Pmp	LMB	RBss	BBul
Lake Trout	0.07	0.11	-0.08	0.08	0.02	0.00	-0.20	-0.27	0.18	-0.09	0.08	-0.07	-0.08	0.12	-0.05
LNSucker		0.28	0.22	-0.02	0.00	-0.05	-0.09	-0.08	0.13	-0.03	-0.08	-0.03	-0.02	-0.03	-0.04
Whitefish			-0.01	0.22	0.10	0.03	0.08	0.11	-0.02	-0.04	-0.05	-0.11	-0.10	-0.03	-0.09
Brook Trout				-0.09	-0.06	-0.11	-0.17	-0.17	0.39	-0.06	-0.12	-0.05	-0.05	-0.05	-0.04
Burbot					0.15	0.02	0.00	0.16	-0.04	0.01	0.01	-0.07	-0.02	-0.01	-0.05
Cisco						0.16	0.30	0.25	0.05	0.02	0.04	-0.03	0.01	-0.02	0.06
Yellow Perch							0.20	-0.01	-0.01	-0.04	0.10	0.24	0.20	0.03	0.18
Pike								0.42	-0.12	0.07	-0.12	0.01	0.09	-0.12	0.09
Walleye									-0.15	0.16	-0.14	-0.11	-0.08	-0.16	-0.06
White Sucker										-0.07	-0.17	-0.05	-0.08	-0.08	-0.05
Redhorse											-0.03	-0.04	-0.04	-0.04	-0.04
SM Bass												0.31	0.18	0.30	0.12
Pumpkinseed													0.27	0.08	0.27
LM Bass														0.06	0.26
Rock Bass															0.03
Brown Bullhead															

Supplementary Table 3. Boosted regression tree (BRT) model results, for species with one or more significantly negative species associations corrected for null expectation (Supplementary Table 2). The results from the association analysis cannot distinguish between species interactions versus habitat affinity – the BRT analysis tests the relative influence (%) of 22 possible drivers of variation affecting the presence of each species, including species composition (the presence of each of the other species) and abiotic factors including degree days, pH, phosphorus, minimum depth, maximum depth, lake area, Secchi depth, and small prey species richness. Correlation refers to how much a predictor model estimates the variation within the raw data (%). The seven grey-shaded cells are the only species pairings where the explanatory species (top row) was one of the ten strongest factors negatively associated (see Suppl. Fig. 5) with the presence of the response species (vertical column on the left side of the table). The species are organized by temperature affinity: blue = cold, green = cool, red = warm.

Focal species	lake whitefish	cisco	lake trout	burbot	brook trout	walleye	pike	small mouth bass	yellow perch	rock bass	white sucker	brown bullhead	pumpkin seed	large mouth bass	Degree days	pH	Total P	Depth min (m)	Depth Max (m)	Area (ha)	secchi depth	small prey richness	No. of trees included in final model	Correlation Between observed and predicted response variables
lake whitefish		20	0.02	15	0	5	0.3	0.1	2	0.02	1	0	0.2	1	22	1	4	1	2	24	1	1	750	76
cisco	21		0	0.10	1	0.10	8	0.03	3	0.05	0.01	0.3	0.10	0	7	3	3	7	5	28	4	10	1000	77
lake trout	0	0		0.01	1	5	1	0	0.01	0	1	0	0.03	0	3	2	9	46	24	2	4	0.3	1050	85
burbot	20	0	0		0.1	0.01	1	0.3	0.3	0.1	0.1	0	0.02	0.1	4	3	1	7	34	23	3	2	900	77
brook trout	0	0.05	6	1		11	11	5	56	0.1	0	0	0	0	3	1	0.2	3	1	0.5	1	1	350	81
pike	1	4	3	0.2	11	24		0.10	22	0.5	0.4	1	0.10	0.2	12	3	2	4	2	6	3	1	1400	82
walleye	3	0	5	0	0.01		36	4	0.07	0.5	1	0	0.10	0.03	12	1	4	2	2	10	1	2	1000	79
small mouth bass	0.02	0.2	0.3	2	3	5	0.2		1	8	0.00	2	10	0.02	39	1	1	4	3	11	4	10	1050	80
yellow perch	2	2	0.2	1	44	0.2	14	3		1	0.2	0.01	0.06	0	10	2	4	4	3	6	2	3	1600	86
rock bass	0	4	0.3	0.2	0.3	2	1	10	3		0	0.05	14	4	39	3	2	3	3	6	3	4	1450	79
white sucker	7	0	5	0	0.2	4	0	0	0.4	0		0	3	0.3	31	2	1	4	3	37	6	1	300	49
brown bullhead	0.1	0.02	0	0.05	0.04	0	2	1	0.01	0.1	0		17	8	60	1	3	1	0.4	1	5	0.3	700	79
Pumpkin seed	4	0	0	0	0.1	0.1	0	2	0.02	4	0	11		11	61	1	1	2	1	1	2	1	650	81
large mouth bass	0	0	0	2	0	0	0	0	0	2	3	6	7		73	2	2	2	2	3	0	0	550	83

Supplementary Table 4. Environmental range comparisons of the negatively associated species pairs identified in Supplementary Table 2. Species AB refers to lakes where both species co-occur. Ten pairs had <u>range overlap</u> in environmental factors yet rarely co-occur. Nine pairs had <u>range compression</u> in lakes of co-occurrence, where the range of environmental conditions in lakes where a species occurs in the absence of the other is wider than when the two species co-occur. BRT refers to pairs where the presence of one species significantly and directly predicts the absence of another (see Suppl. Table 3, Suppl. Fig. 5). Cold water species, cool water species, warm water species - most rarely co-occurring pairs or those with compressed co-occurrence involve species of different temperature groupings.

species pair	% lakes co-occur	RANGE OVERLAP* *Species A vs Species B (ANOSIM)*	RANGE COMPRESSION† *Species A vs AB (PERMDISP)*	RANGE COMPRESSION *Species B vs AB (PERMDISP)*	BRT *The presence of A predicts B and vice versa*	CO-OCCURRENCE RANKING
Brooktrout/Pike	0.50%	0.138 (0.02%)	ns	ns	Yes	*species rarely co-*
Brooktrout/Walleye	0.30%	0.150 (0.02%)	ns	ns	Yes	*occurring (Fig.*
Brooktrout/SMBass	0.60%	0.193 (0.02%)	ns	ns	Yes	*2A)*
SMBass/LongnoseSucker	3.50%	0.203 (0.02%)	ns	ns		
Brooktrout/Burbot	1.40%	0.293 (0.02%)	ns	ns		
Brooktrout/Brownbullhead	1.08%	0.361 (0.02%)	ns	ns		
Brooktrout/LakeTrout	4.80%	0.381 (0.02%)	ns	ns	Yes	
LMBass/Whitefish	3.10%	0.448 (0.02%)	ns	ns		
Brooktrout/LMBass	0.15%	0.565 (0.02%)	NA^	NA^		
LakeTrout/LMBass	4.50%	0.641 (0.02%)	ns	ns		
LakeTrout/Pike	27.90%	0.318 (0.02%)	ns	compressed (p<0.001)	Yes	*one or both*
Pike/Rockbass	36.40%	0.383 (0.02%)	ns	compressed p=0.049)		*species occur in a*
Pike/SMBass	38.20%	0.389 (0.02%)	ns	compressed (p=0.008)		*compressed*
LakeTrout/Pumpkinseed	12.20%	0.475 (0.02%)	ns	compressed (p<0.001)		*range of*
LakeTrout/Walleye	23.70%	0.287 (0.02%)	compressed (p=0.01)	compressed (p<0.001)	Yes	*environments*

Rockbass/Whitefish	23.30%	0.305 (0.02%)	compressed (p<0.001)	compressed (p=0.006)		*when co-*
Burbot/Pumpkinseed	9.70%	0.348 (0.02%)	compressed (p=0.01)	compressed (p<0.001)		*occurring,*
Whitefish/Pumpkinseed	10.50%	0.363 (0.02%)	compressed (p=0.021)	compressed (p<0.001)		*compared to*
LakeTrout/ShrtnoseRedhorse	3.70%	0.428 (0.02%)	compressed (p=0.003)	compressed (p=0.017)		*when found alone*
						(Fig. 2B)
Pike/Whitesucker	71.50%	0.223 (0.10%)	ns	ns		*no compression*
Pumpkinseed/Walleye	7.90%	0.276 (0.02%)	ns	ns		*of environmental*
Rockbass/Walleye	36.50%	0.326 (0.02%)	ns	ns		*lake conditions*
LMBass/Walleye	12.20%	0.376 (0.02%)	ns	ns		*when together*
SMBass/Walleye	39.60%	0.407 (0.02%)	ns	ns		*compared to*
SMBass/Whitesucker	45.10%	0.448 (0.02%)	ns	ns		*when alone (Fig.*
Whitesucker/Rockbass	41.14%	0.428 (0.02%)	ns	ns		*2C)*
Brownbullhead/Whitefish	6.30%	0.425 (0.02%)	ns	ns		
LMBass/Whitesucker	12.90%	0.448 (0.02%)	ns	ns		
LongnoseSucker/Pike	7.60%	0.030 (3.2%)	expanded (p<0.001)	ns		*species is found*
Brooktrout/YellowPerch	2.20%	0.104 (0.3%)	expanded (p=0.04)	ns	Yes	*in a wider range*
LongnoseSucker/Walleye	6.90%	0.123 (0.5%)	expanded (p=0.017)	ns		*of environmental*
WhiteSucker/Walleye	69.20%	0.311 (0.02%)	expanded (p=0.036)	ns		*conditions when*
						the other is
						present (Fig. 2D)

* Overlap refers to the degree of overlap in the multivariate environmental conditions of lakes where Species A and Species B occur without each other - R values closer to 0 indicate greater similarity in multivariate environmental conditions. Numbers in brackets are the percentage of 5000 randomizations for which an R value as high or higher occurred (i.e., a R value equal or greater to the calculated value less than 5% of the time is considered significant).

†Compression refers to a significant change in the multivariate spread or range of environmental conditions, with ‘compressed’ meaning that the range is NARROWER when the species is in the presence of the other species compared to when alone; ‘expanded’ means the range is WIDER for lakes with both species present compared to when the species is alone

^ The PERMDISP test is not applicable (NA) in this case, because only 1 lake contained both species.

Supplementary Figure 1: Bivariate relationships (with linear fits where significant) between total fish diversity and major explanatory drivers of fish occurrences in lakes. These graphs reveal the potential influence of each explanatory factor on fish.

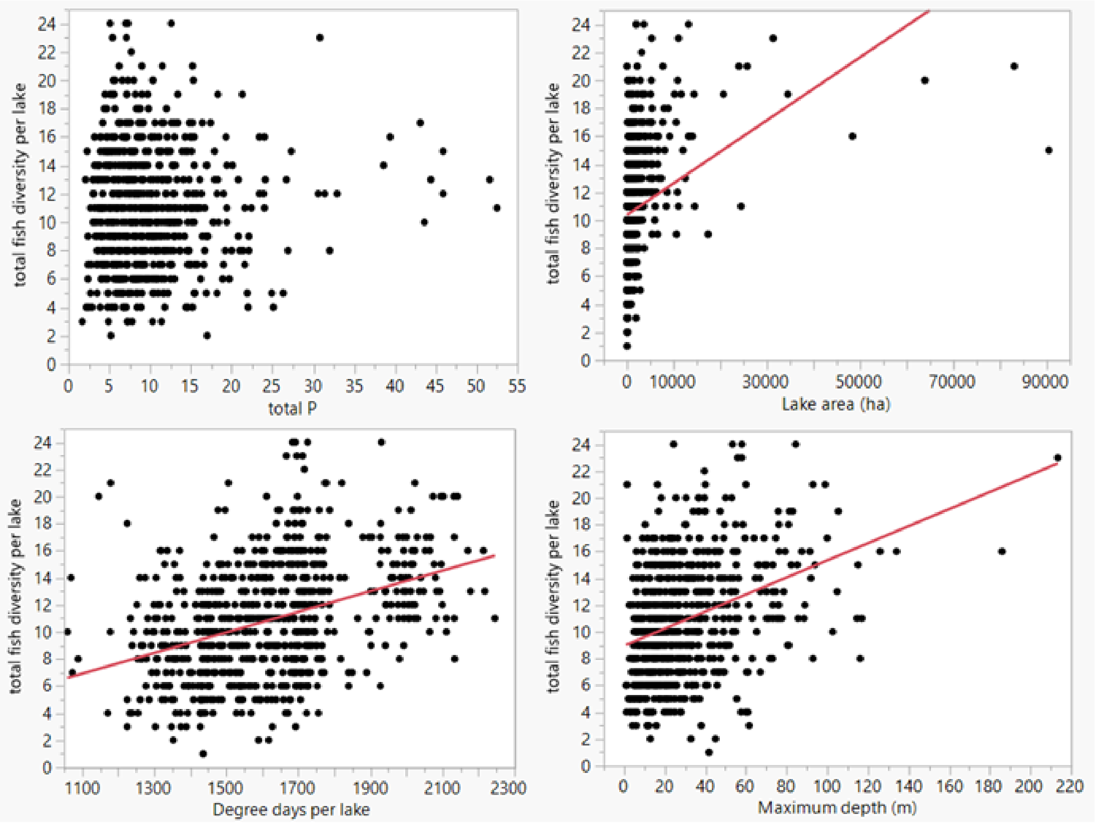

Supplementary Figure 2: Bivariate relationships between abiotic factors in 721 lakes. All relationships were significant with linear fits shown, except latitude despite the concentration of human populations in the southern regions of Ontario. Again, these graphs reveal how any given combination of bivariate factors can significantly interact in lakes, suggesting that integrative analyses testing all simultaneously should better capture how they affect each other, and by extension, the fish communities found within.

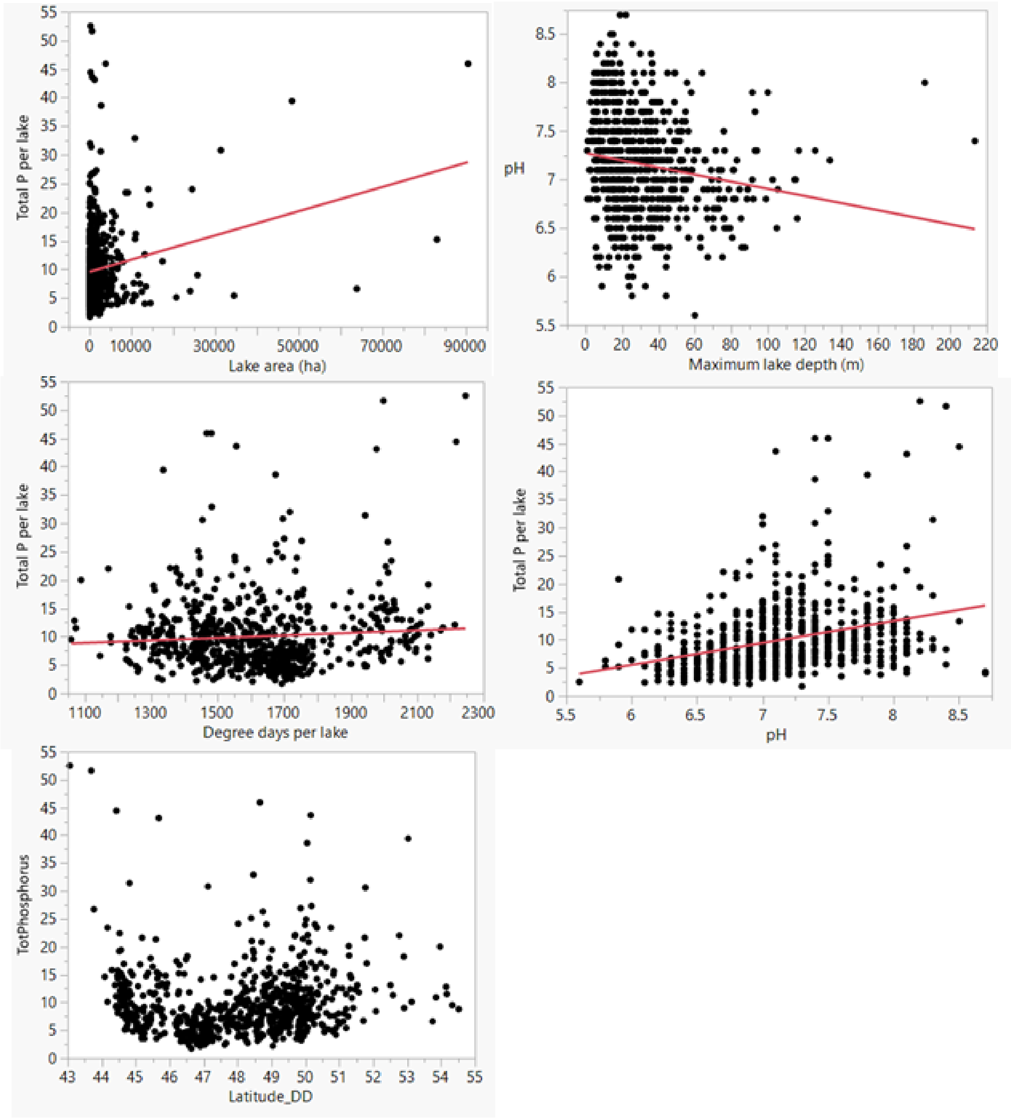

Supplementary Note 1 – Structural Equation Modeling

Our model construction for the SEM analysis began with the entire presence/absence dataset, for 22 fish species in 721 lakes with ten associated environmental measures relating to climate (degree days), lake morphometry (area [ha], maximum depth [m], mean depth [m]), and lake water quality (total P concentration, ph, conductivity, dissolved solids, Secchi depth). To minimize strong co-linearity in our model and maximize parsimony, we screened the correlation matrix among the environmental variables (e.g., see some correlations displayed in Suppl. Fig. 2). This allowed us to reduce the number of environmental variables in the SEM from 10 to 5, with these 5 factors corresponding to our three hypothesized abiotic-based models of influence: climate (degree days), lake morphometry (lake area, maximum lake depth), and water quality (P, pH). All SEM analyses were conducted with R 3.1.2 (R Core Team 2016), using the lavaan package version 0.5-20[4].

For the 'biotic' components of the SEM, we broadly classified the 22 large-sized species (average length > 10 cm) into three functional groups based on habitat affinity and foraging strategy as described in Holm et al.[1] – major predators, littoral species, and pelagic species (Suppl. Table 1). We also grouped the 28 small-sized species (mean length < 10 cm) into a single functional group "small prey species", as described above in the Methods. We tested for influences on total numbers of species per functional group ('functional diversity') and also shifts in species composition (see Methods). For the latter (composition shifts), we identified which functional groups in the SEM were associated with significant species composition changes among lakes using PCA analyses on Hellinger transformed fish data (see Suppl. Fig. 3 and Methods for details). That is to say, which species were significantly associated with the presence of other species. Finally, to satisfy the SEM assumption of multi-normality we log-transformed variables with skewed distributions.

Our SEM analysis was based on the recognized combined importance of abiotic and biotic factors in shaping fish diversity in freshwater lakes[5]. Starting from our initial model that incorporated all levels of interaction among degree days, measures of lake morphometry and water quality, and numbers of species per major functional group, we used modification indices[4] to guide decisions about adding missing paths to the model. We used the “modindices” function from the lavaan package[4], which provides a list of all missing linkages in the model and the expected effect of additions on model fit (Maximum likelihood estimator [ML_{EST}]). We used the modification indices incrementally, adding one path at a time, until no modification indices were higher than 2. After this process, we scanned path regressions and incrementally pruned the less significant ones until there was no more gain in model fit (decreasing ML_{EST}[6]). Our final model converged after 171 iterations (n = 648, ML_{EST} = 4.91, Degree of freedom = 13, *P*=0,977).

Our SEM analysis focused on the hypothesis that an integrated model capturing abiotic and biotic interactions within and among functional groups would best capture the regulation of species richness[5]. Yet, there is also evidence in the fish literature of more singular models shaping the regulation of richness, specifically (I) top-down regulation by major predators, (II) bottom-up models where richness in lower levels of the food chain influences diversity levels above, and (III) abiotic-based models where climate and resources primarily affect species richness at all trophic levels. To further disentangle the relative contribution of abiotic and biotic associations, we decomposed our ‘Integrative’ model into its different components each representing a contrasting hypothesis on the regulation of fish diversity (i.e., no biotic associations between functional groups, top-down associations only, bottom-up associations only).

To make our models comparable we used the same variable structure for all candidate models but altered the direction of effects among functional groups. The models of ‘Top-down’ effects of

consumers on prey (Suppl. Fig. 4b) and the ‘Bottom-up’ effects of prey on consumers (Suppl Fig. 4c) were started from an initial model including all possible top-down or bottom-up associations among fish functional groups. The abiotic factors were then added using modification indices and the less significant regression paths were pruned (using a similar approach as described above). The model without biotic associations (thereafter ‘Abiotic only’ model) included no effects (arrows) between functional groups so that only direct effects of environmental factors were possible (Suppl Fig. 4a). We compared the four models using Akaike Information Criterion (see Suppl. Table 5 and Suppl. Fig. 4). By examining our main ‘Integrative’ model against its reduced components, we were interested in contrasting the magnitude of each factor in the SEM (effect size) versus its contribution to the overall fit of the model (e.g., some factors might have large effect size, but not contribute much to our overall understanding of the system).

Results from our model comparison indicate that the model including only abiotic factors performed the weakest, suggesting that despite being important drivers in terms of effect size, abiotic factors are insufficient to explain the system (i.e., lowest model fit, Suppl. Table 5). That is to say, ignoring biotic associations among fish functional groups led to a significant decline in analytical power (Suppl. Table 5). Alternatively, top-down factors had smaller effect sizes compared to some abiotic factors yet strongly contributed to model fit. This contribution centered largely on two top-down associations: from ‘predator PCA1’ to ‘small prey richness’ and from ‘predator richness’ to ‘pelagic richness’. Removing either of those associations from the ‘Top-Down’ model lead to a jump in the Maximum Likelihood estimator of 40 and 30 points respectively, sending the model at the bottom of the ranking in the AICc comparisons. Overall, the ‘Integrative' model balanced the factors with the greatest effect sizes while maximizing model fit, including the described top down

associations, the strong effects of degree days and lake morphometry, and the bottom-up effect of littoral richness on predator richness (Fig. 1).

The R code for the SEM analysis is provided in full, in Supplementary Note 4.

Supplementary Table 5. Comparison of our best model with more simple alternative hypotheses.

Model	K	AICc	Δ AICc	Difference support
Top-down	48	40119.91	0.00	
Integrative	50	40120.85	0.94	None
Bottom-up	52	40130.71	10.80	Strong
Abiotic	57	40135.64	15.73	Strong

Critical values for model difference support are: **none** ΔAICc < 2; **substantial** 4 < ΔAICc < 7; and **strong**: ΔAICc > 10[7].

Supplementary Figure 3. PCA analysis of Hellinger transformed species associated with significant shifts in species composition in the SEM model (Fig. 1), divided into three functional groups based on broad lake habitat associations. The mechanisms underlying the association shifts cannot be determined by the SEM, and may possibly reflect species interactions, habitat affinity not relating to species interactions, or some combination of the two. All subsequent analyses tested the likelihood of interactions versus habitat in explaining these association shifts, targeting these 16 species with the greatest vector-length separation out of the initial 22 species used in the study.

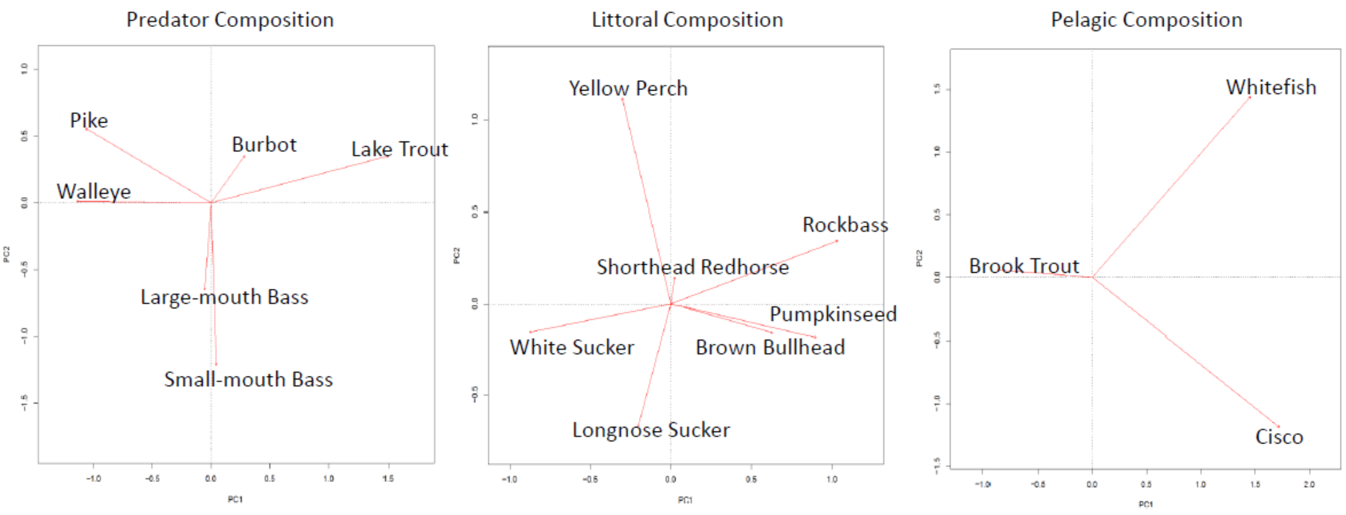

Supplementary Figure 4. Visual representation of all the alternative hypotheses considered in this study to explain fish functional group richness and compositional shifts. Each model was built iteratively with modifications indices[4] starting from an initial model which structure was constrained by specific rules as a function of the hypothesis tested (see Supplementary Notes 1). The a) Abiotic only model represents the effects of abiotic factors of fish functional group richness and composition in the absence of any biotic associations (n = 648, ML_{EST} = 3.03, Degree of freedom = 6, *P*=0.805), b) Top-down model represents the effects of abiotic factors interacting with top-down associations on richness and composition (n = 648, ML_{EST} = 8.66, Degree of freedom = 15, *P*=0.895), c) Bottom-up model represents the effects of abiotic factors interacting with bottom-up associations on richness and composition (n = 648, ML_{EST} = 10.04, Degree of freedom = 11, *P*=0.527), and d) Integrative model represents the combined effects of abiotic factors, bottom-up and top-down associations on richness and composition (n = 648, ML_{EST} = 4.91, Degree of freedom = 13, *P*=0,977).

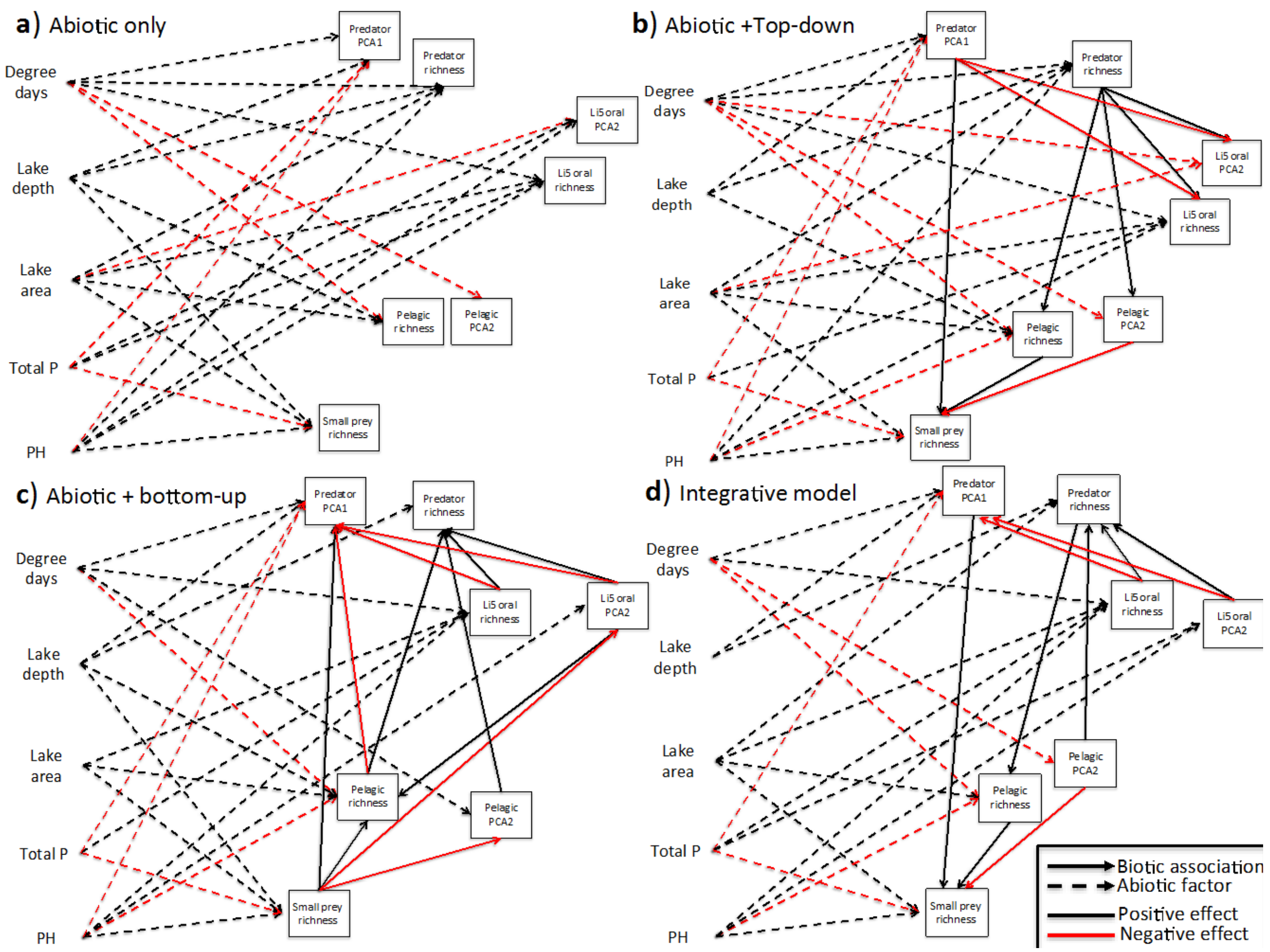

a) Abiotic only
Degree days
Lake depth
Lake area
Total P
PH
Predator PCA1
Predator richness
Li5 oral PCA2
Li5 oral richness
Pelagic richness
Pelagic PCA2
Small prey richness
b) Abiotic +Top-down
Degree days
Lake depth
Lake area
Total P
PH
Predator PCA1
Predator richness
Li5 oral PCA2
Li5 oral richness
Pelagic PCA2
Pelagic richness
Small prey richness
c) Abiotic + bottom-up
Degree days
Lake depth
Lake area
Total P
PH
Predator PCA1
Predator richness
Li5 oral richness
Li5 oral PCA2
Pelagic richness
Pelagic PCA2
Small prey richness
d) Integrative model
Degree days
Lake depth
Lake area
Total P
PH
Predator PCA1
Predator richness
Li5 oral richness
Li5 oral PCA2
Pelagic PCA2
Pelagic richness
Small prey richness
Biotic association
Abiotic factor
Positive effect
Negative effect

Supplementary Note 2 - Individual fish species boosted regression tree (BRT) models

We developed BRT models[8] to assess the relative importance of 22 biotic and abiotic predictor variables for explaining the variation in fish species presence in lakes for 14 of the fish species, i.e. brown bullhead, pumpkin seed, walleye, lake whitefish, pike, cisco, yellow perch, rock bass, white sucker, large-mouth bass, lake trout, small-mouth bass, burbot, and brook trout. In the model developed for each species, we included presence data for the other 13 species to look for possible species relationships, as well as a biotic predictor of the species richness of small prey and seven abiotic characteristics of the lakes, i.e. degree days, pH, phosphorous levels, minimum depth of water, maximum depth of water, area of the lake, and Secchi depth.

All models were fitted in R statistical computing program V. 3.2.1 using the gbm package version 2.1.1[9]. BRT models are simple classification or rule-based models that partition observations into groups based on similar values in the response variables using binary splits that are based on predictor variables[10]. The boosting algorthim then iteratively develops a final model using a forward stage-wise approach that progressively adds trees to the model. BRT models are advantageous for analyzing our data because they can be used to assess different types of predictor variables in the same model, which fits our questions well[11]. We used a tree complexity rate of two (which allows for possible two way interactions) and a learning rate of 0.05 to allow for a suitable predictive performance[10]. Because response variables were based on presence and absence data from surveys conducted within each of the lakes we used a Bernoulli distribution. We show the relative influence of each of the 22 possible predictors of variation in the presence of the 14 fish species in Suppl. Table 3. We also show the number of trees included in the final model and estimates of the correlation between the observed and predicted response variables. Variation in the presence of the different fish species predicted by each BRT model is shown in Suppl. Figure 5, where values are predicted for each

variable by keeping all other variables at mean values.

Supplementary Figure 5: The BRT tests if the occurrence of a species was associated with significant negative changes in the occurrence of the target species, versus all abiotic factors used in our analyses. Only two target species – brook trout (A) and lake trout (B) showed negative relationships versus the occurrence of other species, indicated by the red boxes. These data are the same as shown in Suppl. Fig. 3. Negative relationships are indicated by declining slopes, with the x-axis ranging from 0 (absent) the 1 (present). For the remaining 14 species tested with the BRT (Suppl. Fig. 3), all major drivers of occurrence were abiotic.

(A)

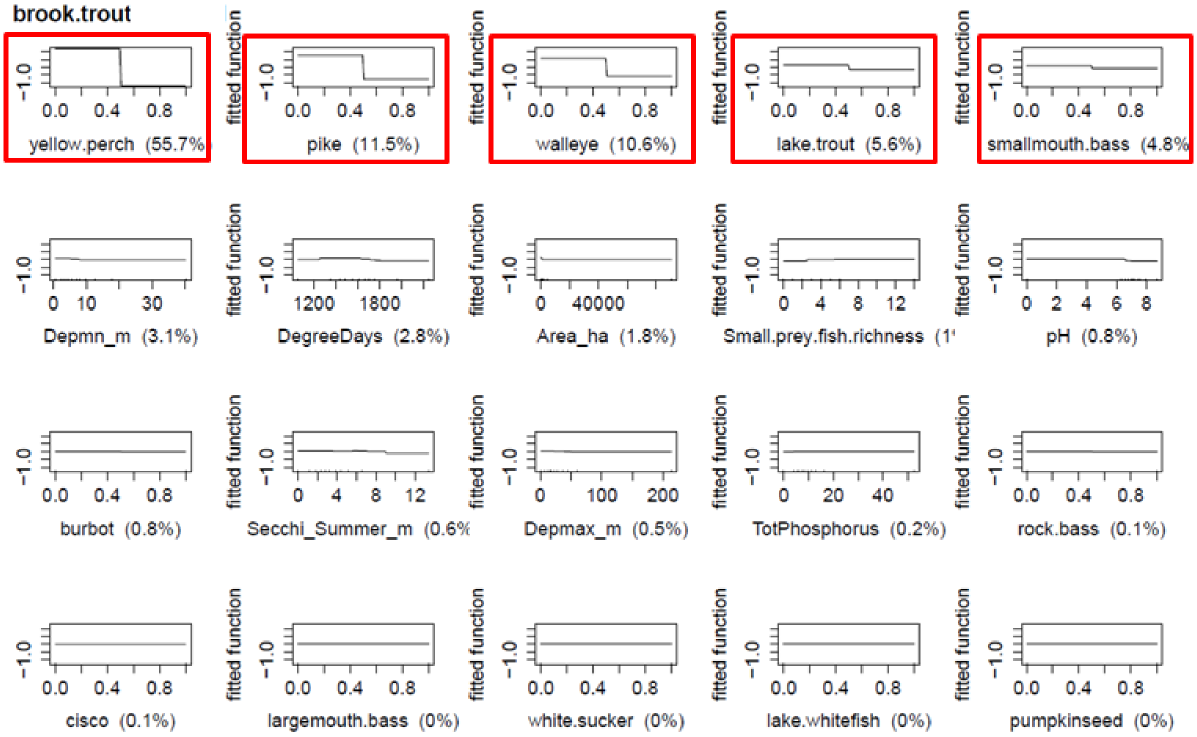

(B)

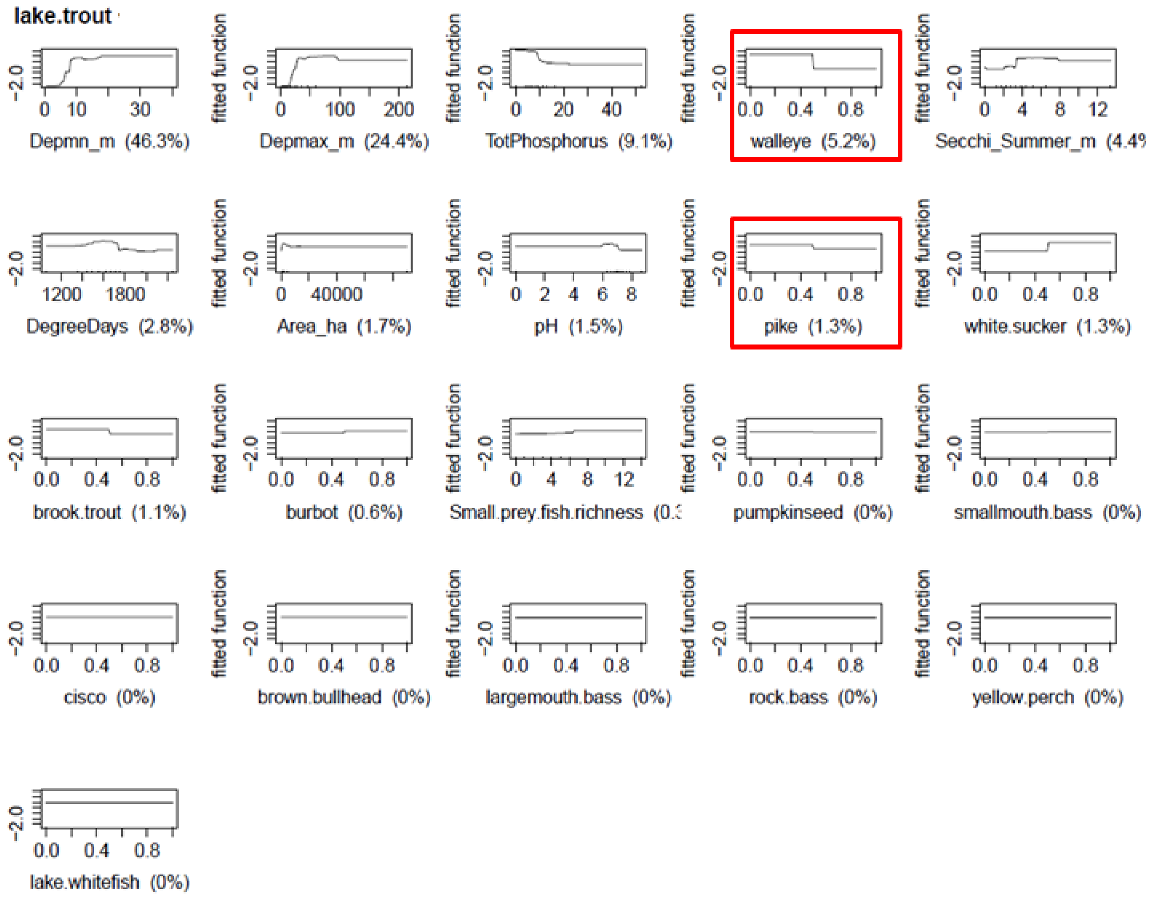
lake.trout
fitted function
-2.0
Depmn_m (46.3%)
Depmax_m (24.4%)
TotPhosphorus (9.1%)
walleye (5.2%)
Secchi_Summer_m (4.4%
DegreeDays (2.8%)
Area_ha (1.7%)
pH (1.5%)
pike (1.3%)
white.sucker (1.3%)
brook.trout (1.1%)
burbot (0.6%)
Small.prey.fish.richness (0.3
pumpkinseed (0%)
smallmouth.bass (0%)
cisco (0%)
brown.bullhead (0%)
largemouth.bass (0%)
rock.bass (0%)
yellow.perch (0%)
lake.whitefish (0%)

Supplementary Note 3 - Multivariate analyses of environmental conditions for species pair combinations

For all species pairs, we calculated the Euclidian distance between three lake combinations (Species A only, Species B only, both) based on seven of the measured abiotic/biogeographic factors that were minimally inter-correlated. These factors are lake area, maximum depth of the lake, summer Secchi depth, conductivity, pH, total phosphorus, and degree days. We eliminated lakes for which one or more of these factors were not measured, leaving 649 of 721 lakes. We log transformed and normalized all variables before the analysis.

To determine the overall degree of overlap in environmental space between lakes with species A only versus lakes with species B only, we used an ANOSIM analysis[12]. This analysis tests the null hypothesis that there are no differences in terms of multivariate environmental conditions, and is equivalent to a non-parametric MANOVA, with permutation to test for significance[12]. The test computes the R statistic, which ranges from 0 (complete overlap in environmental space) to 1 (complete separation in environmental space). As recommended by Clarke et al. (2014)[12], we considered the pairwise test between lakes with species A only versus lakes with species B only after confirming that the global ANOSIM test (considering all lake types: species A only, species B only, both A and B, and neither) was significant. This was true in all cases. In all cases we used 5,000 random permutations to test for significant differences.

If a difference in multivariate environmental conditions was found via the ANOSIM analysis above, it could be due to a shift in location (multivariate average of environmental conditions) and/or a difference in dispersion (multivariate variation or range of environmental conditions). To test for the latter possibility, we determined for each species pair whether lakes with both species co-occurring had a reduced range of environmental conditions compared to lakes with species A only or species B

only. This could happen, for example, if a subordinate competitor or prey species can only co-occur with a dominant competitor or predator in deep and large lakes, with those lake conditions allowing the subordinate more possibilities to persist. Relatedly, we would predict that the subordinate could occur in smaller and shallower lakes, if the dominant species is absent from those lakes. To do this, we used a test of difference in multivariate dispersions (PERMDISP[13]). This is a multivariate extension of Levene's test, measuring the average distance from each lake to the centroid of the group of lakes in the same category, and comparing this average distance between different categories[14]. If coexistence of species A and B is limited to a smaller range of environmental conditions than lakes inhabited by species A without species B, then we would expect to find a significant decline in multivariate dispersion of lakes with both species compared to lakes with species A only. This test also uses permutation to determine significance of the test. We again set the number of permutations to 5,000. We note that for species pairs where the number of lakes containing both species was very small, our power to detect differences in multivariate dispersion was likely limited. For both the ANOSIM and PERMDISP analyses we used the program PRIMER[14]. These tests are also available in the 'vegan' package in R, but R does not allow pairwise tests in the ANOSIM function.

To visualize the similarity among lakes in terms of their environmental conditions, we used PCA to ordinate all lakes in environmental space. We used the 'vegan' package in R to create ordination plots, with vectors to visualize the correlation of each environmental factor with the first two PCA axes.

Supplementary Note 4 – R code for SEM analysis

```
#........................................#
#..........BSM project (SEM)...............#
#........................................#
```

```
#.............................................................................................................................
#
#... Collaborators: Andrew MacDougall et al#
#
#... Author of the script: Eric Harvey (http://ericharvey.weebly.com/)
#
#... Creation date: January 05 2016
#
#... Latest edits: November 07 2016
#
#.............................................................................................................................
#
#... Clear variables
rm(list=ls())
#... Directories paths
datapath = "~/Documents/Research/1.Projects/3.BSM_project/3.Analysis/"
#...Load needed packages
library(vegan)
library(ade4)
library(lavaan)
library(corrplot)
#... Load the data
setwd(datapath)
presence.dat = read.delim("BSM_presence.txt")
presence.dat = na.omit(presence.dat)
#... Identify species of interest for each functional group
predator.of.interest =
c("walleye","lake.trout","pike","smallmouth.bass","largemouth.bass","burbot")
bentho.pelagic.species = c("cisco","brook.trout","round.whitefish","lake.whitefish")
bentho.litoral.species =
c("shorthead.redhorse","silver.redhorse","goldeye","longnose.sucker","white.sucker","br
own.bullhead","channel.catfish","yellow.bullhead","black.crappie","bluegill","pumpkinsee
d","rock.bass","yellow.perch","mooneye","fallfish")
small.prey.species = c("blackchin.shiner","blacknose.shiner","bluntnose.minnow",
"brook.stickleback","central.mudminnow","common.shiner",
"creek.chub","deepwater.sculpin","eastern.blacknose.dace",
"emerald.shiner","fathead.minnow","finescale.dace",
"golden.shiner","Iowa.darter","johnny.darter","lake.chub",
"logperch","longnose.dace","mimic.shiner","Mottled.Sculpin","ninespine.stickleback",
"northern.redbelly.dace","Pearl.dace","sculpin.spp","Slimy.Sculpin",
"spoonhead.sculpin","spottail.shiner","trout.perch")
#... Generate presence/absence matrix with species of interest
pred.comp.mat = presence.dat[,predator.of.interest]
pelagic.comp.mat = presence.dat[,bentho.pelagic.species]
litoral.comp.mat = presence.dat[,bentho.litoral.species]
small.prey.comp.mat = presence.dat[,small.prey.species]
##########################################################################
####################################
```

```
#...Extract variables for SEM
#.....Ordinations analyses for compositional shifts
#.......Change in species composition with PCA
pred.PCA = rda(decostand(pred.comp.mat,"hell"))
pelagic.PCA = rda(decostand(pelagic.comp.mat,"hell"))
litoral.PCA = rda(decostand(litoral.comp.mat,"hell"))
#..........PCA results
pred.PCA
cumsum(pred.PCA$CA$eig/sum(pred.PCA$CA$eig)*100)
pelagic.PCA
cumsum(pelagic.PCA$CA$eig/sum(pelagic.PCA$CA$eig)*100)
litoral.PCA
cumsum(litoral.PCA$CA$eig/sum(litoral.PCA$CA$eig)*100)
#..........PCA diagnosis plots
source ('http://www.davidzeleny.net/anadat-r/doku.php/en:numecolr:cleanplot.pca?
do=export_code&codeblock=0')
cleanplot.pca(pred.PCA)
cleanplot.pca(pelagic.PCA)
cleanplot.pca(litoral.PCA)
dev.off()
#define "evplot" function first:
source ("http://www.davidzeleny.net/anadat-r/doku.php/en:numecolr:evplot?
do=export_code&codeblock=0")
# select the data frame with eigenvalues of particular axes:
ev.pred <- pred.PCA$CA$eig
ev.pelagic <- pelagic.PCA$CA$eig
ev.litoral <- litoral.PCA$CA$eig
# calculate axis-importance and draw the barplots:
evplot (ev.pred)
evplot (ev.pelagic)
evplot (ev.litoral)
dev.off()
#.....Create variables for SEM
#.......Axes representing changes in species composition
pred.PCA1 = scores(pred.PCA)$sites[,1]
pred.PCA2 = scores(pred.PCA)$sites[,2]
pelagic.PCA1 = scores(pelagic.PCA)$sites[,1]
pelagic.PCA2 = scores(pelagic.PCA)$sites[,2]
litoral.PCA1 = scores(litoral.PCA,choices=c(1:4))$sites[,1]
litoral.PCA2 = scores(litoral.PCA,choices=c(1:4))$sites[,2]
#.......Species richness per functional group
pred.richness = specnumber(pred.comp.mat)
small.prey.richness = specnumber(small.prey.comp.mat)
pelagic.richness = specnumber(pelagic.comp.mat)
litoral.richness = specnumber(litoral.comp.mat)
#.......Environmental variables
#..........Geo
Latitude = presence.dat$Latitude_DD
```

```
Longitude = presence.dat$Longitude_DD
DD = presence.dat$DegreeDays
#..........Morphology
Area = presence.dat$Area_ha
Perimeter = presence.dat$Perimeter_km
Depth = presence.dat$Depmn_m
Depth.max = presence.dat$Depmax_m
#..........Productivity
Secchi = presence.dat$Secchi_Summer_m
Conductivity = presence.dat$Conductivity..uS.cm.s.
TDSolids = presence.dat$TDSolids_mgL
TotPhosphorus = presence.dat$TotPhosphorus
PH = presence.dat$pH
##########################################################################
####################################
#...Correlations among variables
#Among biotic factors
biotic.cor =
cor(cbind(litoral.PCA1,litoral.PCA2,pelagic.PCA1,pelagic.PCA2,pred.PCA1,pred.PCA2,
pred.richness,litoral.richness,pelagic.richness,small.prey.richness))
corrplot(biotic.cor,method="number",type="full")
#Among environmental variables
env.cor =
cor(na.omit(cbind(Latitude,Longitude,DD,Area,Perimeter,Depth,Secchi,Conductivity,TD
Solids,TotPhosphorus,PH)))
corrplot(env.cor,method="number",type="full")
##########################################################################
####################################
#...Data frame for SEM ananalysis
#........variables are log transformed if necessary
#........variables need to be all at the same variance scale - to achieve this some are
divided/multiplied by a factor of 10 or 100
data.mod = data.frame(DD/100,Latitude,
Area/1000,Perimeter/100,Depth,
Secchi,TotPhosphorus,Conductivity/10,PH,TDSolids/10,
as.numeric(pred.richness),as.numeric(small.prey.richness),pelagic.richness,litoral.richne
ss,
pred.PCA1*100,pred.PCA2*100,pelagic.PCA1*100,pelagic.PCA2*100,litoral.PCA1*100,
litoral.PCA2*100)
colnames(data.mod) = c("DD","Latitude",
"Area","Perimeter","Depth",
"Secchi","TotPhosphorus","Conductivity","PH","TDSolids",
"pred.richness","small.prey.richness","pelagic.richness","litoral.richness",
"pred.PCA1","pred.PCA2","pelagic.PCA1","pelagic.PCA2","litoral.PCA1","litoral.PCA2")
#........Correlation matrix of the data
round(cov2cor(cov(data.mod)),3)
##########################################################################
####################################
```

```
#...Structural equation models
#Each model is built by starting from a simplified model from which paths are sequentially added based on the modification indices until no modification indices are >2.
#The initial simplified model for each alternative model is built as follow:
#Model 1 - Abiotic factors only
#Model 2 - Top-down regulation: Initial model was only top-down interactions and then abiotic factors are added sequentially
#Model 3 - Bottom-up regulation: Initial model was only bottom-up interactions and then abiotic factors are added sequentially
#Model 4 - Integrative model (main model from the paper): Abiotic + Top-down + Bottom-up (based on our intial meta-model - see Supp.Mat)
#If the modification indice proposed to add a path that is forbidden according to the model (e.g., between trophic groups in the abiotic model), the proposition is ignored and the next best proposition is implemented.
#If the modification indice value of adding a path between an abiotic factor and a functional group is the same as the value of adding a path between said trophic group and another trophic group, then it was interpreted as a signal that the effect of said abiotic factor might be mediated by a trophic interaction
library(semTools)
#.......Model 1 --> Model with only abiotic factors
BSM.mod1 <- "
# Regression
small.prey.richness ~ DD + PH + Area + TotPhosphorus + Depth
pelagic.richness ~ DD + PH + Depth + Area + TotPhosphorus
pelagic.PCA2 ~ DD + TotPhosphorus
litoral.richness ~ DD + Area + TotPhosphorus + PH + Depth
litoral.PCA2 ~ DD + Area + PH + TotPhosphorus
pred.richness ~ PH + Depth + Area + DD
pred.PCA1 ~ DD + PH + TotPhosphorus + Depth
"
BSM.mod1.fit = sem(BSM.mod1,data=data.mod) #run the SEM
BSM.mod1.fit #visualize fitting results
summary(BSM.mod1.fit,stand=T,rsq=T) #visualize path coefficients
modindices(BSM.mod1.fit) #modification indices
# To inspect model estimates and parameters
varTable(BSM.mod1.fit) #Variance table
inspect(BSM.mod1.fit,"cov.ov") #co-variance matrix
inspect(BSM.mod1.fit,"est") #regression estimates
#
#.......Model 2 Top-down regulation --> paths only from higher to lower trophic groups + abiotic factors
#Notice that Depth - a factor that was very important in Model 1 is now mainly important for top-predators - it suggests that the effect of depth on other groups is mediated by top-predators
BSM.mod2 <- "
# Regression
small.prey.richness ~ pred.PCA1 + pred.richness + pelagic.richness + pelagic.PCA2 +
```

```
litoral.richness + PH + Area + TotPhosphorus
pelagic.richness ~ pred.richness + DD + Depth + PH + Area + TotPhosphorus +
pred.PCA1
pelagic.PCA2 ~ pred.richness + litoral.richness + DD
litoral.richness ~ pred.richness + pred.PCA1 + DD + TotPhosphorus + Area + PH
litoral.PCA2 ~ pred.richness + pred.PCA1 + DD + Area + pelagic.PCA2
pred.richness ~ PH + Depth + Area + DD
pred.PCA1 ~ DD + PH + TotPhosphorus + Depth
# Residual correlations
pred.richness ~~ pred.PCA1
pelagic.richness ~~ pelagic.PCA2
litoral.richness ~~ litoral.PCA2
"
BSM.mod2.fit = sem(BSM.mod2,data=data.mod)
BSM.mod2.fit
summary(BSM.mod2.fit,stand=T,rsq=T)
modindices(BSM.mod2.fit)
# To inspect model estimates and parameters
varTable(BSM.mod2.fit) #Variance table
inspect(BSM.mod2.fit,"cov.ov") #co-variance matrix
inspect(BSM.mod2.fit,"est") #regression estimates
#
#.......Model 3 Bottom-up regulation --> Ppaths only from lower to higher trohpic groups+
abiotic factors
BSM.mod3 <- "
# Regression
small.prey.richness ~ PH + Depth + Area + TotPhosphorus
pelagic.richness ~ small.prey.richness + DD + Depth + PH + Area + litoral.PCA2
pelagic.PCA2 ~ small.prey.richness + litoral.richness + Area + DD + TotPhosphorus
litoral.richness ~ small.prey.richness + DD + TotPhosphorus + Area + PH
litoral.PCA2 ~ small.prey.richness + DD + PH
pred.richness ~ small.prey.richness + pelagic.richness + pelagic.PCA2 + litoral.richness
+ litoral.PCA2 + Depth + DD + Area + TotPhosphorus
pred.PCA1 ~ small.prey.richness + pelagic.richness + pelagic.PCA2 + litoral.richness +
litoral.PCA2 + Depth + DD + TotPhosphorus + PH
# Residual correlations
pred.richness ~~ pred.PCA1
pelagic.richness ~~ pelagic.PCA2
litoral.richness ~~ litoral.PCA2
pelagic.richness ~~ litoral.richness
"
BSM.mod3.fit = sem(BSM.mod3,data=data.mod)
BSM.mod3.fit
summary(BSM.mod3.fit,stand=T,rsq=T)
modindices(BSM.mod3.fit)
# To inspect model estimates and parameters
varTable(BSM.mod3.fit) #Variance table
inspect(BSM.mod3.fit,"cov.ov") #co-variance matrix
```

```
inspect(BSM.mod3.fit,"est") #regression estimates
#
#.......Model 4 Integrative model (main model from our meta-model) --> Abiotic factors +
top-down + bottom-up
BSM.mod4 <- "
# Regression
small.prey.richness ~ PH + Area + TotPhosphorus + pred.PCA1 + pelagic.richness +
pelagic.PCA2
pelagic.richness ~ DD + PH + Depth + Area + TotPhosphorus + pred.richness
pelagic.PCA2 ~ DD + TotPhosphorus + pred.richness
litoral.richness ~ DD + Area + TotPhosphorus + PH + pred.richness
litoral.PCA2 ~ DD + Area + PH + TotPhosphorus
pred.richness ~ PH + Depth + litoral.richness + litoral.PCA2 + pelagic.richness +
pelagic.PCA2 + TotPhosphorus + small.prey.richness + Area
pred.PCA1 ~ DD + PH + TotPhosphorus + Depth + litoral.richness + litoral.PCA2
# Residual correlations
litoral.richness ~~ litoral.PCA2
pelagic.richness ~~ pelagic.PCA2
pred.richness ~~ pred.PCA1
litoral.richness ~~ small.prey.richness
"
BSM.mod4.fit = sem(BSM.mod4,data=data.mod)
BSM.mod4.fit
summary(BSM.mod4.fit,stand=T,rsq=T)
modindices(BSM.mod4.fit) #modification indices
resid(BSM.mod4.fit,type="raw")
# To inspect model estimates and parameters
varTable(BSM.mod4.fit) #Variance table
inspect(BSM.mod4.fit,"cov.ov") #co-variance matrix
inspect(BSM.mod4.fit,"est") #regression estimates
#
#######################
#Model goodness of fit and comparaison
#######
#AIC comparaisons
library(AICcmodavg)
aictab(list(BSM.mod1.fit,BSM.mod2.fit,BSM.mod3.fit,BSM.mod4.fit),c("Abiotic","TD","BU
","Integrative"))
# Model selection based on AICc:
#
# K AICc Delta_AICc AICcWt Cum.Wt LL
# TD 48 40119.91 0.00 0.61 0.61 -20008.03
# Integrative 50 40120.85 0.94 0.38 1.00 -20006.15
# BU 52 40130.71 10.80 0.00 1.00 -20008.72
# Abiotic 57 40135.64 15.73 0.00 1.00 -20005.22
#After removing 'Predator PCA1 --> 'Small prey richness' from the 'Top-down' model
# Model selection based on AICc:
#
```

```
# K AICc Delta_AICc AICcWt Cum.Wt LL
# Integrative 50 40120.85 0.00 0.99 0.99 -20006.15
# BU 52 40130.71 9.86 0.01 1.00 -20008.72
# Abiotic 57 40135.64 14.79 0.00 1.00 -20005.22
# TD 47 40157.95 37.10 0.00 1.00 -20028.22
#After removing 'Predator richness' --> 'Pelagic richness' from the 'Top-down model'
# K AICc Delta_AICc AICcWt Cum.Wt LL
# Integrative 50 40120.85 0.00 0.99 0.99 -20006.15
# BU 52 40130.71 9.86 0.01 1.00 -20008.72
# Abiotic 57 40135.64 14.79 0.00 1.00 -20005.22
# TD 47 40150.85 30.00 0.00 1.00 -20024.66
#Observations on AIC comparisons
# 1. Abiotic only is last
# 2. The difference between TD and ALL is not substantial
# 3. The difference between TD/ALL and BU/Abiotic is substantial
# 4. It is important to note that no interactions in the TD model does not indicate
of actual top-down interactions (none of them are negative!) except for one strong topdown
interaction between top-predators and small prey richness
# 5. In the context of observation #4 - It seems that the only reason why BU does not
perform well is because it is lacking the very important top-down effect of top-predators
on small prey richness
# 6. Observation #5 can be verified easily by removing the top-down path from toppredators
to small prey richness in the top-down model ( 'Predator PCA1' --> 'Small
prey richnes':ML increases by 40 points and the P-value = 0.0000, 'Predator richness' --
> 'Pelagic richness': ML increase 33 points) - therefore the good fit of the dop-down
model is driven by this keystone interaction in the model
#Conclusion:
# 1. A mixture of all variables is essential to understand the system. Both bottom-up
and top-down effects are very important, and in many case they seem to mediate the
effect of abiotic factors (e.g., Lake depth).
# 2. Thus, Model 4 is confidently the best model to understand the system because it
integrates all information and the mediated effects in the most parsimonious way
# 3. Model 4 could be analyzed further to gain better insights about the system.
#END
#####
```

Supplementary References

1. Holm, E., Mandrak, N. E., & Burridge, M. E. (2010). Freshwater fishes of Ontario. *Royal Ontario Museum, Toronto*.

2. Hasnain, S. S., Shuter, B. J., & Minns, C. K. (2013). Phylogeny influences the relationships linking key ecological thermal metrics for North American freshwater fish species. *Canadian Journal of Fisheries and Aquatic Sciences*, *70*, 964-972.
3. Legendre, P., and de Cácares, M. 2013. Beta diversity as the variance of community data: dissimilarity coefficients and partitioning. Ecology Letters, 16: 951-963.
4. Rosseel, Y. 2012. lavaan: An R package for structural equation modeling. Journal of statistical software 48:1–36
5. Jackson, D. A., Peres-Neto, P. R., & Olden, J. D. (2001). What controls who is where in freshwater fish communities the roles of biotic, abiotic, and spatial factors. *Canadian journal of fisheries and aquatic sciences*, *58*(1), 157-170.
6. Grace, J. B., D. R. Schoolmaster, G. R. Guntenspergen, A. M. Little, B. R. Mitchell, K. M. Miller, and E. W. Schweiger. 2012. Guidelines for a graph-theoretic implementation of structural equation modeling. Ecosphere 3:art73.
7. Burnham, K. P., and D. R. Anderson. 2002. Model Selection and Multimodel Inference: A Practical Information-Theoretic Approach. Springer Science & Business Media.
8. Friedman, J.H. 2001. Greedy function approximation: the gradient boosting machine. Annal Stat. 29:1189-1232.
9. R Development Core Team (2013). R: language and environment for statistical computing. R. F. f. S. Computing. Vienna, Austrria, http://www.R-project.org/.
10. Ridgeway 2015 Package GBM. CRAN http://code.google.com/p/gradientboostedmodels/
11. Elith, J., Kearney, M., & Phillips, S. (2010). The art of modelling range-shifting species. *Methods in ecology and evolution, 1*(4), 330-342.

12. Clarke, K.R., Gorley, R.N., Somerfield, P.J., Warwich, R.M. (2014) Change in marine communities: an approach to statistical analysis and interpretation, 3rd edition. PRIMER-E: Plymouth, UK.

13. Anderson, M.J., Ellingsen, K.E. & McArdle, B.H. (2006) Multivariate dispersion as a measure of beta diversity. Ecology Letters, 9, 683–693.

14. Anderson, M.J., Gorley, R.N. & Clarke, K.R. (2008) PERMANOVA+ for PRIMER: Guide to Software and Statistical Methods. PRIMER-E, Plymouth, UK.
